# Drug-like Antagonists of P2Y Receptor Subtypes: An Update

**DOI:** 10.1021/acs.jmedchem.5c00249

**Published:** 2025-04-28

**Authors:** Mahesh Puthanveedu, Rebecca Knight, Michael J. Stocks

**Affiliations:** † Division of Biomolecular Sciences and Medicinal Chemistry, Biodiscovery Institute, School of Pharmacy, University of Nottingham, Nottingham NG7 2RD, United Kingdom; ‡ Division of Physiology, Pharmacology and Neuroscience, School of Life Sciences, University of Nottingham, Nottingham NG7 2UH, United Kingdom; § Centre of Membrane Proteins and Receptors, University of Birmingham and Nottingham, The Midlands NG7 2UH, United Kingdom

## Abstract

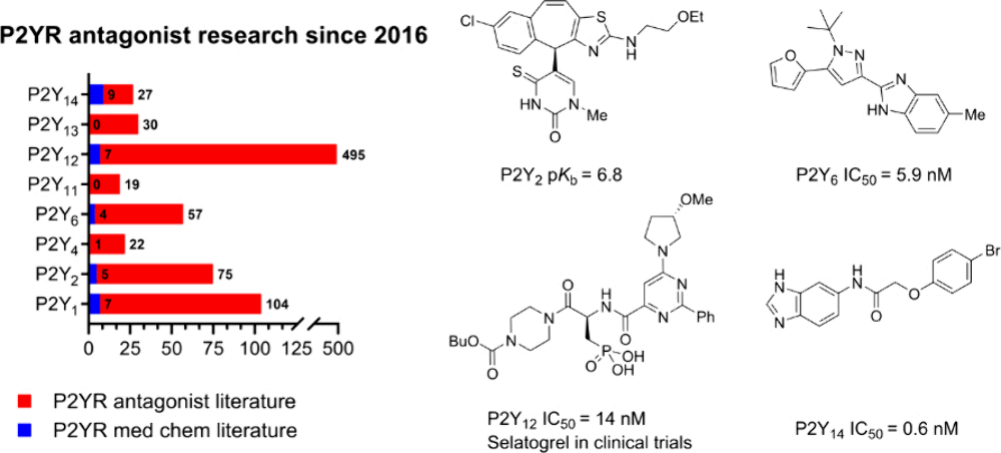

The hunt for drug-like P2YR antagonists continues, stimulated by ever-increasing pharmacological evidence for their clinical benefit and the astonishing array of biological functions which they orchestrate, including platelet aggregation, cancer proliferation, pain, neurodegenerative diseases, and immune regulation. Extensive research has identified modulators of P2Y receptors. However, only a limited number of small-molecule antagonists for the P2Y_12_ receptor have received approval for their clinical use. Recent pioneering discoveries of small-molecule ligand-bound X-ray crystal structures for the P2Y_1_ and P2Y_12_ receptors and homology modeling has stimulated research groups to explore orthosteric and allosteric receptor antagonists, aided in part by the discovery of fluorescent P2YR imaging tools and sensitive screening methods that allow the identification of low affinity P2Y receptor antagonists. This Perspective critically assesses P2Y receptor antagonists published since 2016, highlighting potential oral lead- or drug-like compounds that offer opportunities for the development of molecules for clinical evaluation.

## Significance

- Latest developments around oral drug-like antagonists, their properties, and the clinical significance of P2Y receptors are discussed.
- P2Y_1_R and P2Y_12_R 3D structures have helped to find novel small -molecule ligands for P2Y subtypes, enabling concept testing in animal models.
- Despite promising advances and existing extensive research, no candidates have progressed successfully through clinical trials except for the P2Y_12_R.
- Growing evidence for the application of P2YR antagonists in neuroinflammation, vascular regulation, and the progression of neurodegenerative diseases.

## Introduction

Purinergic receptors mediate the extracellular signaling of nucleotides, such as adenosine-5′-triphosphate (ATP) and uridine-5′-triphosphate (UTP), and thus influence an extensive array of biological functions. Initially, the purinergic receptors were divided into the P1 and P2 receptors due to their activation by adenosine and nucleotides, respectively, as proposed by Burnstock in 1978.^1^ Subsequently, the P2 receptors were further subdivided into P2X ligand-gated cation channels and P2Y G protein-coupled receptors (P2YRs).^1^ The P2YRs are class A rhodopsin-like G protein-coupled receptors (GPCRs), which exhibit seven transmembrane-spanning α-helices with an extracellular *N*-terminus andan intracellular *C*-terminus (unless stated otherwise, henceforth P2YRs refer to the human isoform).^2^ The expression of P2YRs have been reported in most cell and tissue types, with important roles in physiological processes, including inflammation, platelet aggregation, and pain transmission.^1^ However, these receptors have also been implicated in a multitude of pathophysiological conditions, including cancer, Alzheimer’s disease, atherosclerosis, and diabetes.^1^ Therefore, the identification of oral drug-like compounds that target the P2YRs could have therapeutic benefit for various unmet areas of clinical need.

**Figure 1:**
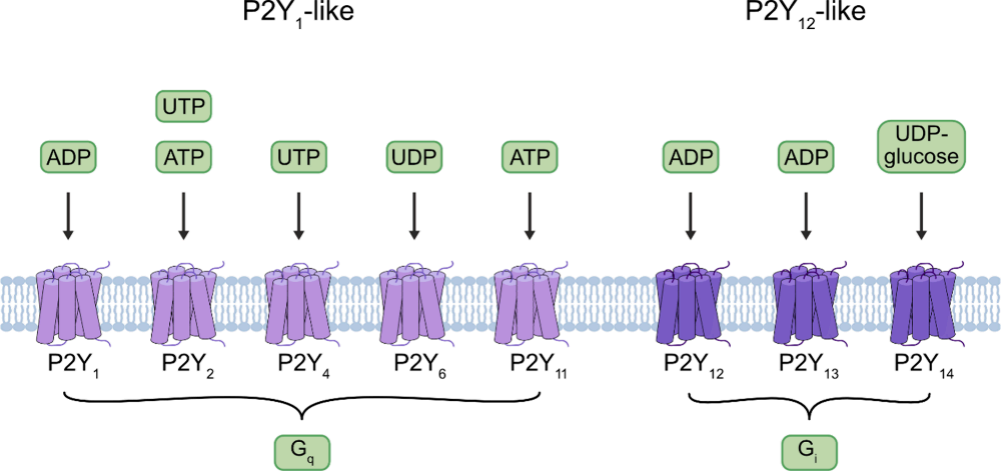
An overview of the P2Y receptor subtypes and their endogenous ligands.

There are eight characterized P2YRs, which are subdivided into two groups based on their preferential G-protein coupling and phylogenetic relationship.^1^ The first “P2Y_1_-like” subgroup contains the P2Y_1,2,4,6,11_ receptors, which share 28–52% sequence homology and are primarily coupled to G_q_, although these receptors can also signal through other G-proteins.^2^ The second “P2Y_12_-like” subgroup contains the P2Y_12,13,14_ receptors, which share 45–50% sequence homology and are G_i_ coupled.^2^ However, P2YRs also differ according to their selectivity for nucleotides. The P2Y_1_, P2Y_12_, and P2Y_13_ receptors are activated by adenosine-5′-diphosphate (ADP), whereas the P2Y_11_ receptor responds to ATP.^2^ The P2Y_2_ receptor is activated by both ATP and UTP.^2^ The P2Y_4_ and P2Y_6_ receptors respond to UTP and uridine-5′-triphosphate (UDP), respectively, while the P2Y_14_ receptor is activated by UDP-sugars, such as UDP-glucose (Figure 1).^2^

Overall, the development of oral drug-like small molecules for the P2YRs has proven challenging because, as a chemical starting point, the endogenous ligands are associated with low bioavailability, metabolic instability, and nonspecific association with biological membranes. This is due to the presence of negatively charged phosphate groups in the nucleotide agonists, which are important for retaining potency in the P2YRs. Furthermore, because several P2YRs respond to the same nucleotide agonist, ensuring subtype selectivity is also difficult. Consequently, many of the reported P2YR antagonists are not viable chemical lead compounds due to poor physicochemical properties, potency, and/or selectivity. Thus, far, the only P2YR antagonists successfully developed for clinical use target the P2Y_12_ receptor (P2Y_12_R) as an antithrombotic treatment, although there are several selective P2YR inhibitors with preclinical data (Table S1). However, within the past decade, the publication of the ligand-bound X-ray crystal structures of the P2Y_1_ and P2Y_12_ receptors has provided opportunities to identify new chemotype P2YR antagonists using structure-based drug design and screening.^3−5^

This Perspective will explore the advances in identifying oral drug-like antagonists for the P2YRs, building on a previous perspective published in this journal almost a decade ago.^6^ For each P2YR, an introduction to the pharmacology and therapeutic benefit for antagonism will be presented, followed by an examination of the reported compounds and their progress toward becoming orally bioavailable drug-like antagonists.

## Antagonists of the P2Y_1_ Receptor

The P2Y_1_ receptor (P2Y_1_R) is G_q_ protein-coupled which, when activated by ADP, stimulates phospholipase C (PLC) mediated cleavage of phosphatidylinositol 4,5-bisphosphate (PIP_2_) into the secondary messenger’s inositol triphosphate (IP_3_) and diacylglycerol (DAG). The secondary messengers IP_3_ and DAG coordinate the release of Ca^2+^ ions from intracellular stores and activate protein kinase C (PKC), respectively.^7^ Coactivation of the P2Y_1_ and P2Y_12_ receptors is necessary for ADP-mediated platelet activation, leading to aggregation and thrombus formation; therefore, the development of selective P2Y_1_R antagonists as antithrombotic agents is a promising and active area of research.^8^ In addition to its role in thrombosis, modulation of the P2Y_1_R has been implicated in various physiological and pathological processes, including inflammation. Activation of the P2Y_1_R on certain immune cells leads to the release of inflammatory mediators, contributing to the overall inflammatory response.^9^

**Figure 2:**
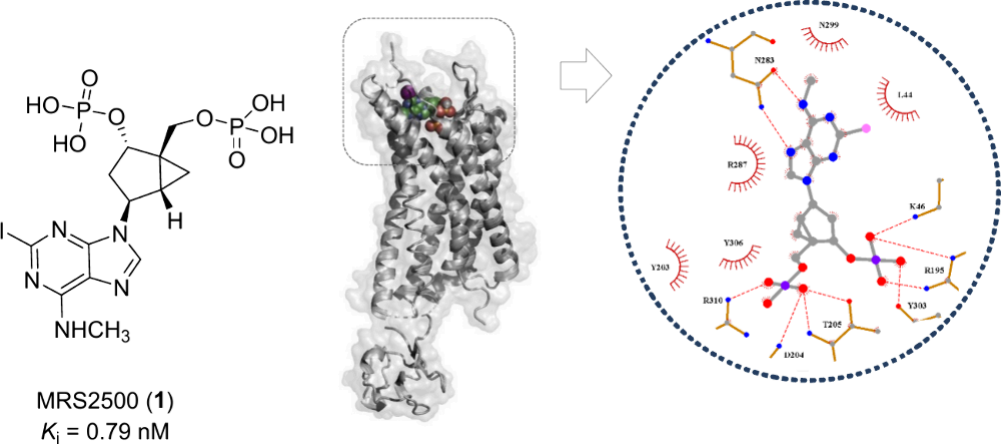
Chemical structure, binding site, and interactions of MRS2500 (**1**) with the P2Y_1_R according to the structure in Protein Data Bank (PDB ID: 4XNW). PyMOL was used for 3D visualization (The PyMOL Molecular Graphics System, ver. 3.0, Schrödinger, LLC), and LigPlot+ was used for 2D interactions.^10^

ADP is the endogenous agonist of the P2Y_1_R, with purified P2Y_1_R exhibiting 100-fold greater affinity for ADP compared to ATP, which serves as a partial agonist for this receptor.^11^ Consequently, initial attempts to develop P2Y_1_R antagonists were focused on the development of nucleotide analogues.^12^ Among them, the antagonist MRS2500 (**1**) containing a *N*-methanocarba ring system is used widely as a pharmacological probe of the P2Y_1_R (Figure 2).^13^ Even though MRS2500 and related analogues show excellent pharmacological properties, such as potent inhibition of ADP-induced aggregation of human platelets, their pharmacokinetic (PK) properties due to the presence of charged phosphate groups at physiological pH make them less attractive as oral drug candidates. However, they are still valuable tool compounds to study receptor pharmacology and aid in new drug development efforts targeting the P2Y_1_R.

**Figure 3:**
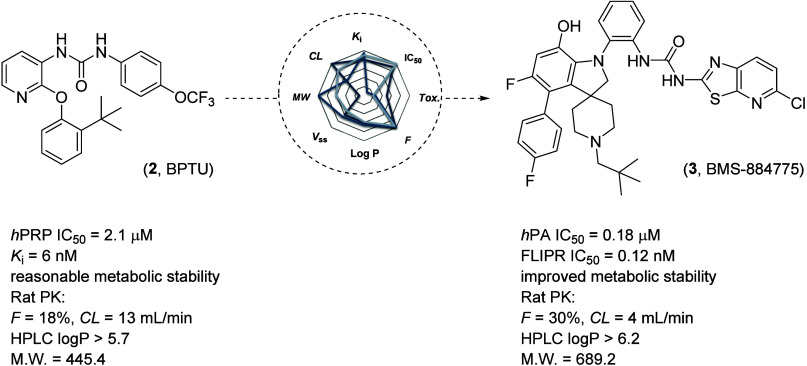
Evolution of P2Y_1_R antagonist BPTU (**2**) to the clinical candidate BMS-884775 (**3**).

In the past decade, there has been a growing interest in P2Y_1_R antagonist development, especially for use as antithrombotics. Scientists within pharmaceutical companies, including Pfizer, GlaxoSmithKline (GSK), and Bristol Myers Squibb (BMS), have carried out high-throughput screening (HTS) of large libraries of compounds to find suitable candidates for drug development. In these efforts they have identified a new diaryl urea chemotype as potent and selective non-nucleotide inhibitors of the P2Y_1_R. Among the non-nucleotide antagonists of the P2Y_1_R with potential antithrombotic effects, *N*-[2-[2-(1,1-dimethylethyl)phenoxy]-3-pyridinyl]-*N*′-[4-(trifluoromethoxy)phenyl]urea (**2**, BPTU) was discovered through optimizing a HTS hit from a BMS compound library and proved an interesting candidate for further optimization. Multi-Parameter Optimization (MPO) of lead compounds during preclinical development is a very important phase in drug discovery. The initial preclinical compounds, in almost all cases, will have issues progressing and require optimization of properties such as solubility, selectivity, and off-target activities. In the case of BPTU, the compound was potent in the P2Y_1_R membrane binding assay (*K*_i_ = 6 nM). However, moderate *in vitro* antiplatelet activity (IC_50_ = 2.1 μM) in the ADP-induced platelet aggregation assay in platelet-enriched human plasma (*h*PRP) was observed. In addition, poor aqueous solubility and low bioavailability (*F* = 18%) precluded BPTU from being progressed further.^14^ Generally, knowledge about how small molecules bind to the receptor is greatly beneficial when balancing drug-likeness and bioactivity in chemical optimization. Unfortunately, the X-ray crystal structure of the P2Y_1_R was not available at the time. Therefore, structure–activity relationship (SAR) studies based on homology models and substitution at different positions on BPTU eventually led to the discovery of compound BMS-884775 (**3**).^15^ BMS-884775 demonstrated excellent *in vitro* potency, selectivity, and desirable properties, such as lower clearance, improved bioavailability, and good metabolic stability across species. In rabbit efficacy/bleeding models, BMS-884775 demonstrated similar antithrombotic efficacy with less bleeding compared to the clinical antithrombotic drug prasugrel (Figure 3).

However, during the MPO of BPTU to BMS-884775, the lipophilicity of the compound increased significantly (clogP 7.72, HPLC logP > 6.2) along with the size of the molecule (MW = 689 Da). During the lead optimization process, in most cases, the potency of the compound was directly attributed to increases in the lipophilicity of the molecule. This resulted in highly potent compounds with extremely poor aqueous solubility (<10 ng/mL for BMS-884775). The undesired physicochemical properties such as the high molecular weight, lipophilicity, and poor aqueous solubility of BMS-884775 resulted in low oral bioavailability in preclinical studies (28% in rats and 6% in monkeys, 5 mg/kg po) when dosed as cosolvent solution formulations.^16^

**Figure 4:**
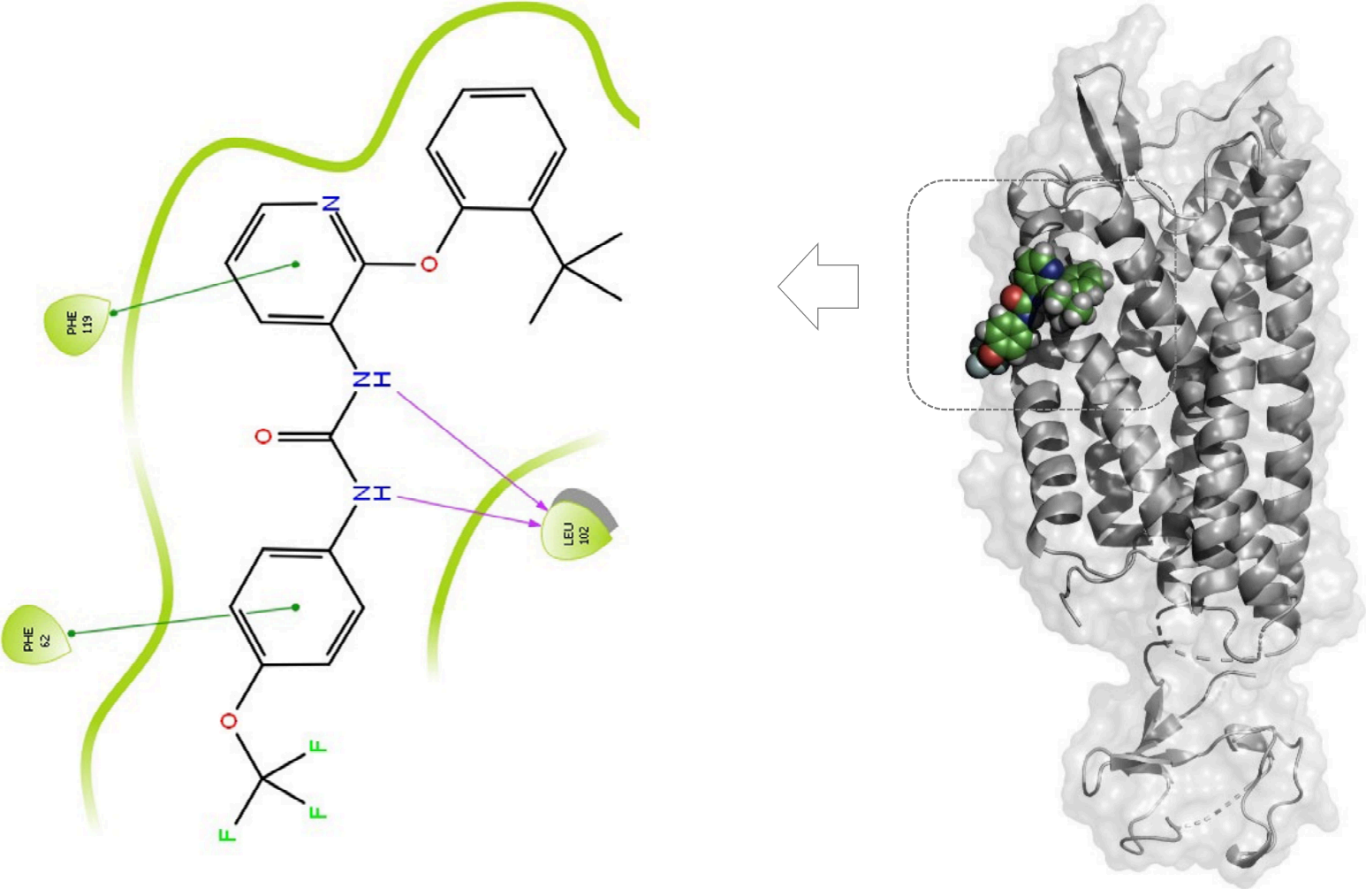
BPTU-P2Y_1_R cocrystal structure (PDB ID: 4XNV) showing BPTU (**2**) binding at the lipidic receptor–membrane interface (right). H-bond interactions and π–π interactions of **2** with the target protein are illustrated on the left. PyMOL visualization was used (The PyMOL Molecular Graphics System, ver. 3.0, Schrödinger, LLC).

Later, when the X-ray cocrystal structure (PDB ID: 4XNV) of the P2Y_1_R in the presence of BPTU was solved, it was found that BPTU binds on the lipidic interface of the transmembrane domain and is an allosteric modulator of the P2Y_1_R (Figure 4). The relatively shallow ligand-binding pocket, formed by aromatic and hydrophobic residues of helices I, II, and III and ECL1, accommodates BPTU predominantly through hydrophobic interactions, which demonstrates why the binding affinity of BPTU correlated directly with HPLC logP values. The P2Y_1_R-MRS2500 X-ray cocrystal structure (PDB ID: 4XNW) was also solved, showing the ligand occupies a pocket within the seven transmembrane bundle, defined by residues mainly from the *N*-terminus, ECL2, and helices VI and VII (Figure 2). The resolved structure demonstrated the involvement of a few key residues such as Tyr203, Tyr306, and Arg310 in the nucleotide antagonist’s binding, as predicted previously through computational and mutagenesis studies.^17^ However, many residues that were speculated to have a direct role in ligand binding were found to be located deeper than the actual MRS2500 binding site. The crystal structures of the P2Y_1_R bound with an orthosteric and allosteric ligand not only provides more information about small-molecule modulation of this receptor but also presents opportunities for modeling other P2Y receptors.^3,18,19^

**Figure 5:**
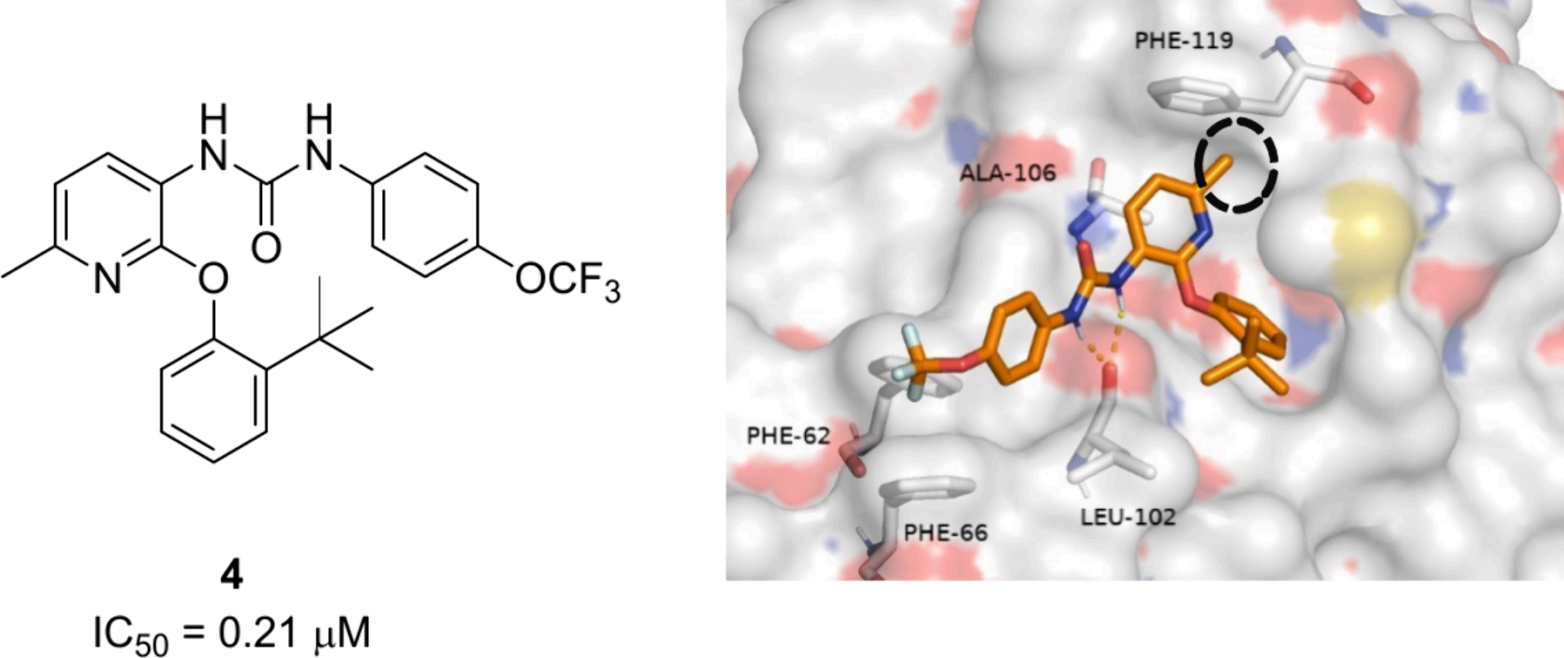
Chemical structure of compound **4**. Diagram showing **4** interacting with a previously unexplored hydrophobic pocket in the P2Y_1_R (right). OpenEye OMEGA^21^ and OE Docking (OEDOCKING 4.3.1.3. OpenEye, Cadence Molecular Sciences, Inc., Santa Fe, NM) applications were used, and PyMOL was used for visualization (The PyMOL Molecular Graphics System, ver. 3.0, Schrödinger, LLC).

Knowledge of the unique binding mode of BPTU helped to design novel analogues that utilized the unexplored regions of the P2Y_1_R binding pocket to generate additional hydrophobic interactions. For instance, among the several analogues synthesized by Peng et al., compound **4**, which contains a methyl group on the pyridine ring, interestingly showed slightly increased the P2Y_1_R antagonistic activity compared to BPTU when tested *in vitro* (1321N1 cells stably expressing the P2Y_1_R) and inhibited ADP-induced platelet aggregation in rats. Docking studies indicated that the methyl group extended to a small hydrophobic pocket that was previously not utilized by BPTU in its binding.^20^ Even though the challenges posed during the lead optimization of BPTU would still be relevant for these analogues, this study reiterates that the availability of a target 3D structure could help in the structure-based drug design of novel potent P2Y_1_R antagonists (Figure 5).

**Figure 6:**
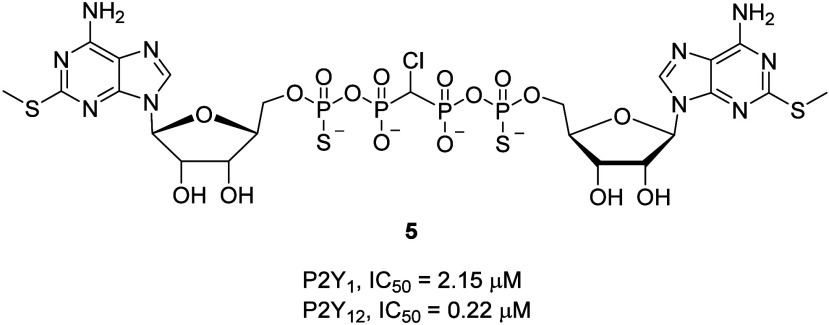
Chemical structure and potency of compound **5**, an Ap_4_A derivative.

The P2Y_1_R initiates platelet response to stimuli, while the P2Y_12_R has a role in the maintenance of the platelet response. Therefore, dual P2Y_1_ and P2Y_12_ receptor antagonism may offer synergistic benefits in antiplatelet therapy.^22^ Dinucleoside polyphosphate analogue **5** designed based on *P*^1^,*P*^4^-di(adenosine-5′) tetraphosphate (Ap_4_A) inhibited ADP-induced platelet aggregation by targeting both the P2Y_1_ and P2Y_12_ receptors (Figure 6).^23,24^ This chemotype, however, might not be suitable for oral administration due to poor physicochemical properties.

**Figure 7:**
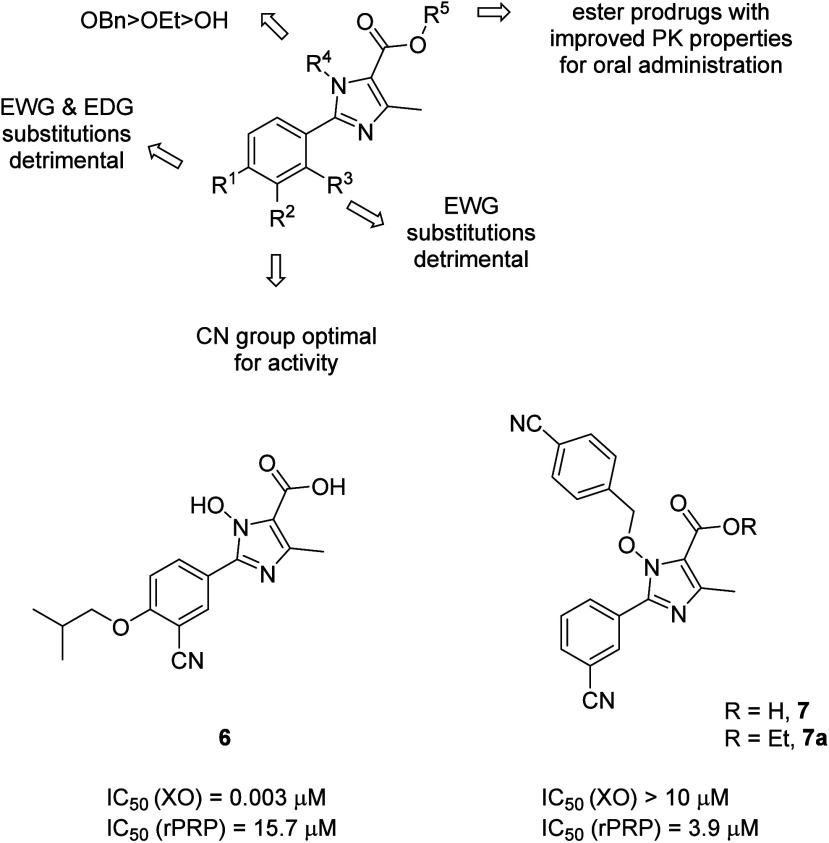
Chemical structures and IC_50_ values of 2-phenylpyrazole derivatives in xanthine oxidase (XO) and in the platelet-rich plasma (PRP) aggregation assay.

Recently, a novel 2-phenyl imidazole-based chemotype of antithrombotic agent was identified from the xanthine oxidase inhibitor **6** (XO IC_50_ = 3.0 nM), with anticoagulative side effects (Figure 7).^25^ Structure activity studies showed that benzylating the hydroxy group at *N*^1^ is optimal for switching the xanthine oxidase activity to antithrombotic activity. The SAR summary for the 2-aryl-imidazole-5-carboxylic acid derivatives evaluated for their P2Y_1,12_R inhibitory activity is depicted in Figure 7. Compound **7**, which had the best potency (IC_50_ = 3.9 μM) and physicochemical properties for oral drug delivery (*t*_1/2_ = 13 h in SD rats, 10 mg/kg po, *t*_max_ = 0.46 h, AUC = 135.08 ng·h/mL, *F* = 32.4%), inhibited both the P2Y_1_ and P2Y_12_ receptors (Table S2). The ethyl ester prodrug (**7a**) of compound **7** inhibited the P2Y_1_R with potency (IC_50_ = 2.6 μM) comparable to that of BPTU as confirmed by measured cytosolic Ca^2+^ levels. Compound **7** also inhibited P2Y_12_R with an IC_50_ of 148.9 μM. Selectivity studies against other purinergic receptors, including P2X and other P2Y subtypes, were not supplied. However, the oral toxicity studies with a 2000 mg/kg single dose of prodrug did not cause any problems in mice. Compound **7**, at 10 mg/kg po, produced *in vivo* antithrombotic efficacy comparable to that of ticagrelor in a FeCl_3_ rat model and presented reduced bleeding risk in a rat tail bleeding model.^26^ Interestingly, the 2-phenyl imidazole scaffold is also reported in the design of FXIa inhibitors with antithrombotic activity.^27^ Computational studies on the binding of **7** with the P2Y_1_R showed multiple interactions with residues in the binding site of the MRS2500-P2Y_1_ complex. This suggests the 2-phenylpyrazole derivative has distinctive binding from that of BPTU. In principle, non-nucleotide antagonists that bind to the P2Y_1_R inside the pocket of the transmembrane helix similarly to MRS2500, rather than at the lipidic interface as with BPTU, could be more hydrophilic and drug-like. Thus, further investigation of the SAR and binding characteristics of compound **7** is warranted.

**Scheme 1:**
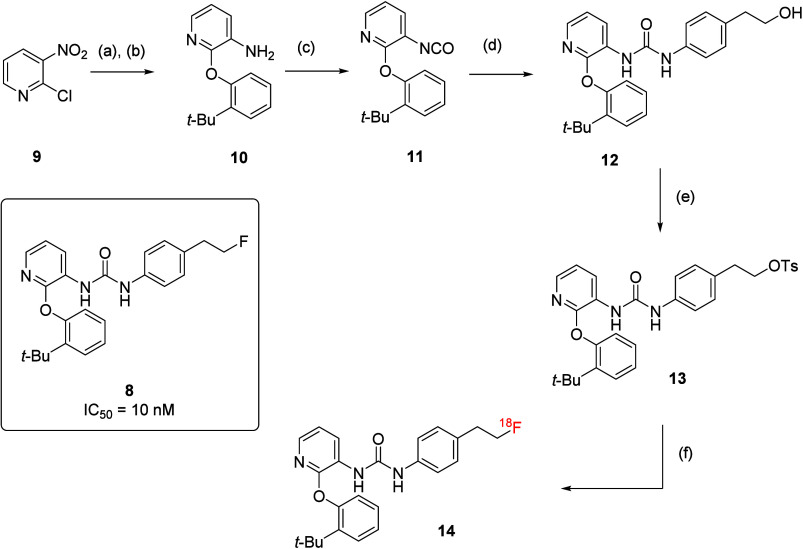
Reagents and conditions: (a) *ortho*-*t*-Bu-phenol, Cs_2_CO_3_, DMF, 70 °C, 14 h; (b) Pd/C, H_2_; (c) diphosgene, Et_3_N, DCM, 0 °C to rt, 2 h; (d) corresponding substituted aniline, toluene, 80 °C, 1 h; (e) TsCl, pyridine, DCM, rt, 16 h; (f) [^18^F] TBAF in *t*-BuOH, 90 °C, 10 min.

The P2Y_1_R plays a crucial role in various brain functions and processes associated with mental and neurological disorders.^28^ However, the current understanding of the P2Y_1_R function in both the healthy and diseased brain is limited due to the lack of P2Y_1_R-selective compounds that can cross the blood–brain barrier (BBB). Initial attempts to develop ^18^F-labeled positron emission topography (PET) tracers based on the P2Y_1_R allosteric modulator BPTU led to the identification of **14**, an ^18^F-labeled analogue of compound **8**. Briefly, base-mediated S_N_Ar at the 2-position of pyridine scaffold **9**, followed by Pd/C hydrogenation, provided the amino intermediate **10**. The corresponding isocyanate **11** was prepared by treating **10** with diphosgene and was further reacted with the aniline intermediate to afford **12**, followed by tosylation to yield **13**. The radio-synthetic process using tetra-*n*-butylammonium [^18^F] fluoride successfully produced the labeled ligand **14** with high radiochemical purity (Scheme 1). Despite the promising initial developments, mouse *in vivo* experiments showed rapid metabolism of **14**, hindering further exploration. Nonetheless, the high potency (IC_50_ = 10 nM for **8**) and unique allosteric binding mode of such compounds present intriguing opportunities for further optimization as P2Y_1_R PET tracers. The successful development of selective receptor tracers holds promise for studying the role and distribution of the P2Y_1_R in the brain, shedding light on its involvement in neurological diseases. The known binding mode and interactions of the BPTU class of compounds with the P2Y_1_R could guide the design in this regard.^29^

P2Y_1_R antagonists inhibit platelet activation. However, the ubiquitous expression of the P2Y_1_R in other tissues renders it questionable as an antithrombotic target,^8^ especially when the P2Y_12_R is expressed nearly exclusively in both platelets and the brain. Emerging research suggests a potential role of purinergic signaling, including the P2Y_1_R, in cancer progression, angiogenesis, and metastasis.^30^ The abundance of the P2Y_1_R in other parts of the body and its role in inflammation, central nervous system (CNS) disorders, autoimmune disorders, and cancer make it a target worth exploring further. In addition, combining P2Y_1_R inhibition with other antiplatelet agents, such as P2Y_12_R inhibitors, could offer a dual antiplatelet therapy approach with a rapid onset of action. The P2Y_1_R has roles in both host defense and hemostasis. Recent studies exploring P2Y_1_R signaling suggest possible biased agonism occurring from activation via other endogenous nucleotides instead of ADP. Therefore, development of compounds eliciting biased anti-inflammatory responses without affecting platelet aggregation is also an exciting but challenging possibility.^31^

## Antagonists of the P2Y_2_ Receptor

The P2Y_2_ receptor (P2Y_2_R) is activated by both ATP and UTP and is principally coupled to the G_q_ protein.^7^ The P2Y_2_R is predominantly expressed in the lungs, female and male tissues, proximal digestive tract, and lymphoid tissues as well as at lower levels in skin and soft tissue.^32^ Inhibition of the P2Y_2_R could have clinical benefits for diseases driven by chronic inflammation, such as atherosclerosis and idiopathic pulmonary fibrosis, as well as cancer, where the receptor has been implicated in tumor growth and metastasis.^33−36^

**Figure 8:**
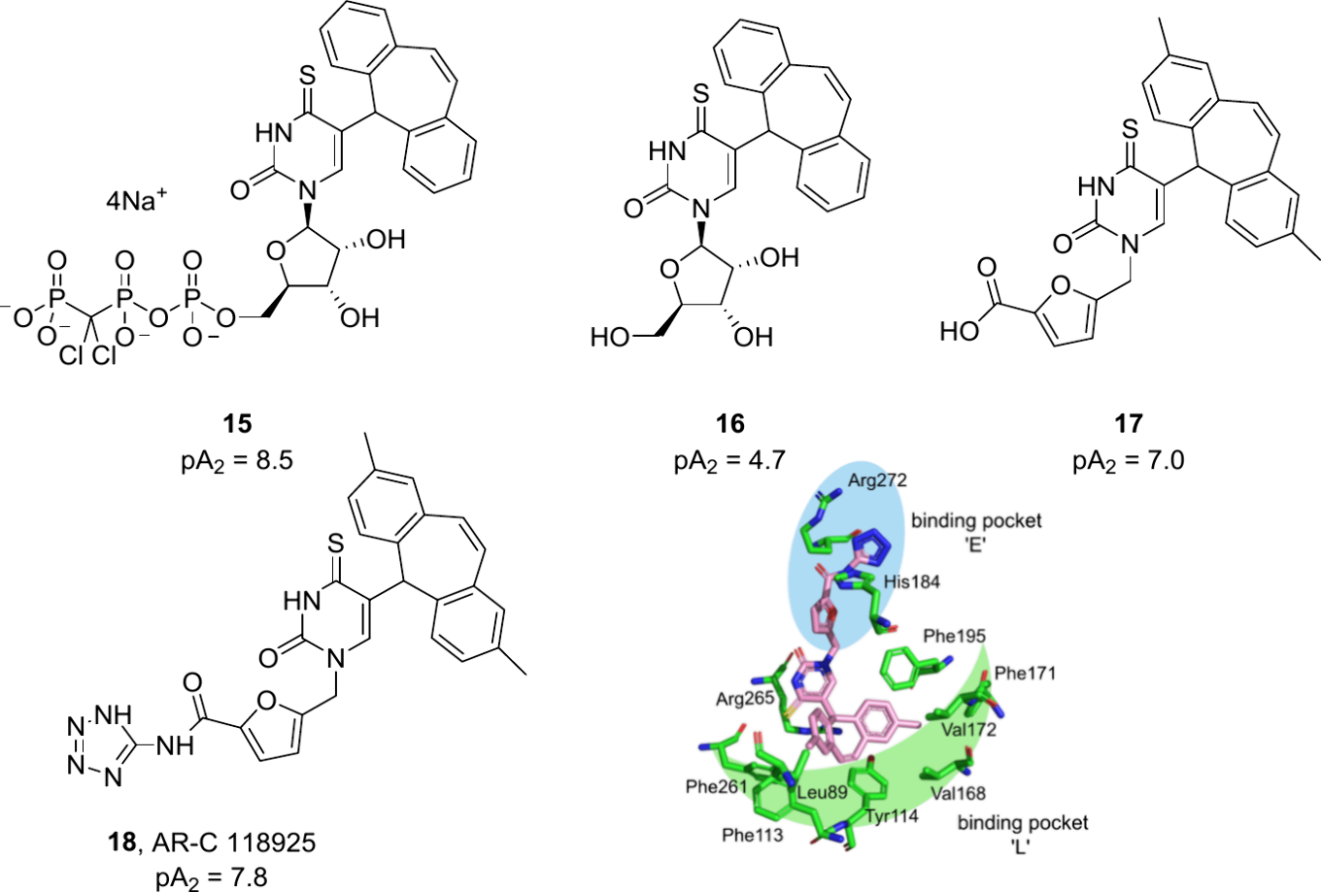
From UTP derivatives to AR-C118925: evolution of P2Y_2_R antagonists. Graphics generated using the PyMOL Molecular Graphics System, ver. 3.0, Schrödinger, LLC.

The only reported P2Y_2_R antagonists developed by an industrial research group were disclosed in a series of patents by AstraZeneca in the 1990s.^37^ More recently, the design and synthesis of one of the most potent and selective of these antagonists, AR-C 118925 (**18**), was published. Potency was determined using a fluorescence-based assay measuring the inhibition of UTP-induced intracellular Ca^2+^ release in Jurkat cells stably transfected with the P2Y_2_R. Using UTP as a starting point, conversion to 4-thiouridine and replacement of the triphosphate βγ-oxygen with dichloromethylene improved stability, while the introduction of a lipophilic group at C-5 of the uridine delivered antagonism of the P2Y_2_R, as highlighted by tricycle **15** (pA_2_ = 8.5). Removal of the triphosphate group, necessitated by the requirement for oral drug-like compounds, significantly reduced the potency (**16**; pA_2_ = 4.7). However, activity was recovered through symmetrical substitution on the tricycle and replacement of the ribose with heteroaromatic rings containing carboxylic groups, with the most potent being furan-containing **17** (pA_2_ = 7.0). The inclusion of the carboxylic acid group was intended to recapitulate interactions of the UTP α-phosphate with the P2Y_2_R, and replacement with an amidotetrazole bioisostere further improved the potency (**18** or AR-C 118925; pA_2_ = 7.8). However, despite being potent and selective, the high lipophilicity of AR-C 118925 (clogP = 5.3) compromises its physicochemical properties and consequently it is not bioavailable when administered orally (Table S2, Figure 8).^38^

The X-ray crystal structure resolved for the P2Y_1_R has subsequently been used to develop a homology model for the P2Y_2_R which, for the binding site amino acids, had equivalent prediction quality to the AlphaFold structure.^3,39,40^ The P2Y_2_R homology model was validated and optimized using site-directed mutagenesis and pharmacological studies; residues in the putative orthosteric site were mutated and the potency of structurally diverse agonists and antagonists determined in fluorescence-based assays measuring intracellular Ca^2+^ release in 1321N1 astrocytoma cells stably transfected with the P2Y_2_R. The predicted binding pose of AR-C118925 in the P2Y_2_R homology model indicated the 2,8-dimethyl-5*H*-dibenzo[*a,d*][7]annulene tricycle is anchored in a lipophilic binding pocket (pocket L) with strong hydrophobic interactions, which could confer its high potency.^39,40^ The furan also occupied a binding pocket near the extracellular lumen (pocket E) with the tetrazolate, which would be deprotonated at physiological pH, forming salt bridges with basic amino acids such as His184 and Arg272.^39,40^ In the P2Y_2_R homology model, these basic amino acids bind the triphosphate of UTP and concurrently, the mutation of His184 or Arg272 to alanine leads to a >100-fold or 350-fold decrease in UTP potency, respectively (Figure 8).^39,41^

**Figure 9:**
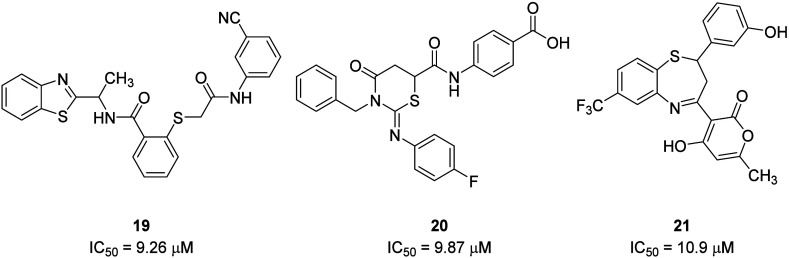
Micromolar hits of structure-based virtual screening using a P2Y_2_R homology model based on the P2Y_1_R X-ray crystal structure.

The optimized P2Y_2_R homology model in complex with AR-C118925 was employed to generate receptor grids for Glide docking-based structure-based virtual screening (SBVS) of 3.2 million compounds. The potency of the initial hit compound analogues was determined using a fluorescence-based assay measuring the inhibition of UTP-induced intracellular Ca^2+^ release in 1321N1 astrocytoma cells stably transfected with the P2Y_2_R. Three P2Y_2_R antagonist scaffolds, which were benzothiazole (*e.g.*, **19**; IC_50_ = 9.26 μM), thiazine (e.g., **20**; IC_50_ = 9.87 μM), and thiazepine derivatives (e.g., **21**; IC_50_ = 10.9 μM), displayed micromolar potency.^40^ The predicted binding pose in the P2Y_2_R homology model suggested these compounds partially occupied pocket L, while the terminal aryl groups in **19** and **20** bound in pocket E and were exposed to the extracellular space, forming interactions with basic amino acids, similarly to the tetrazolate in AR-C118925.^39,40^ While displaying high micromolar affinity and limited elucidated SAR, these compounds could offer starting points for further optimization by rational drug design (Figure 9).

**Figure 10:**
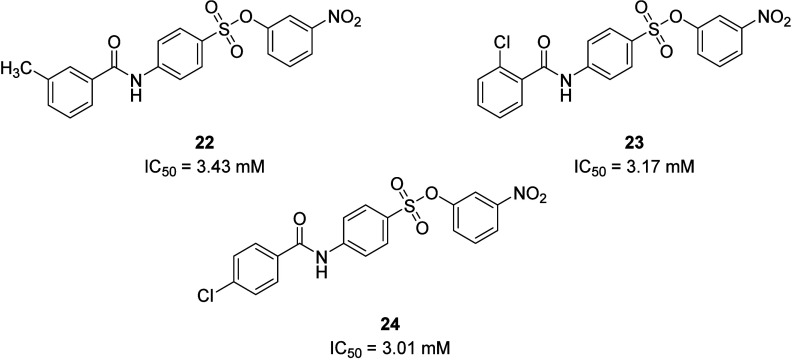
A selection of dual P2Y_2_ and GPR17 antagonist structures.

Exploration of suramin-derivatives as dual antagonists for the P2Y_2_R and the orphan receptor GPR17 identified benzenesulfonates **22** (IC_50_ = 3.43 μM), **23** (IC_50_ = 3.17 μM), and **24** (IC_50_ = 3.01 μM). Potency was determined by using a fluorescence-based assay measuring the inhibition of ATP- or UTP-induced intracellular Ca^2+^ release in P2Y_2_R-1321N1 cells. The sulfonate group was essential for activity, and any replacement with urea, sulfonamide, or carboxamide significantly reduced or abolished potency at the P2Y_2_R. The positioning of the methyl and nitro substituents was also important, as changing from the *meta*- to the *para*-position diminished the potency of **22**. Replacement of the methyl with trifluoromethyl, acetyl, propionyl, or *m*-methoxy also abolished activity; however, the introduction of chlorine in the *ortho*- or *para*-position (**23** and **24**) was tolerated. Compounds **22** and **23** induced a rightward shift in the concentration–response curve of UTP, but **24** reduced the maximal response, suggesting a noncompetitive allosteric binding mode. These compounds also demonstrated selectivity for the P2Y_2_R over the P2Y_4_R (∼30-fold for **22** and **23**, and 3-fold for **24**) and the rat P2Y_6_R (∼30-fold) except for **22**, which had an IC_50_ of 6.2 μM at the rat P2Y_6_R. However, no compounds in this study showed selectivity for the P2Y_2_R over GPR17 and thus further SAR investigation would be required to deliver a sole P2Y_2_R antagonist.^42^ Additionally, drugs containing nitro groups are associated with toxicity due to enzymatic reduction during metabolism.^43^ Nevertheless, the selectivity over the closely related P2Y_4_R and P2Y_6_R could be promising for further optimization by rational drug design (Figure 10).

**Figure 11:**
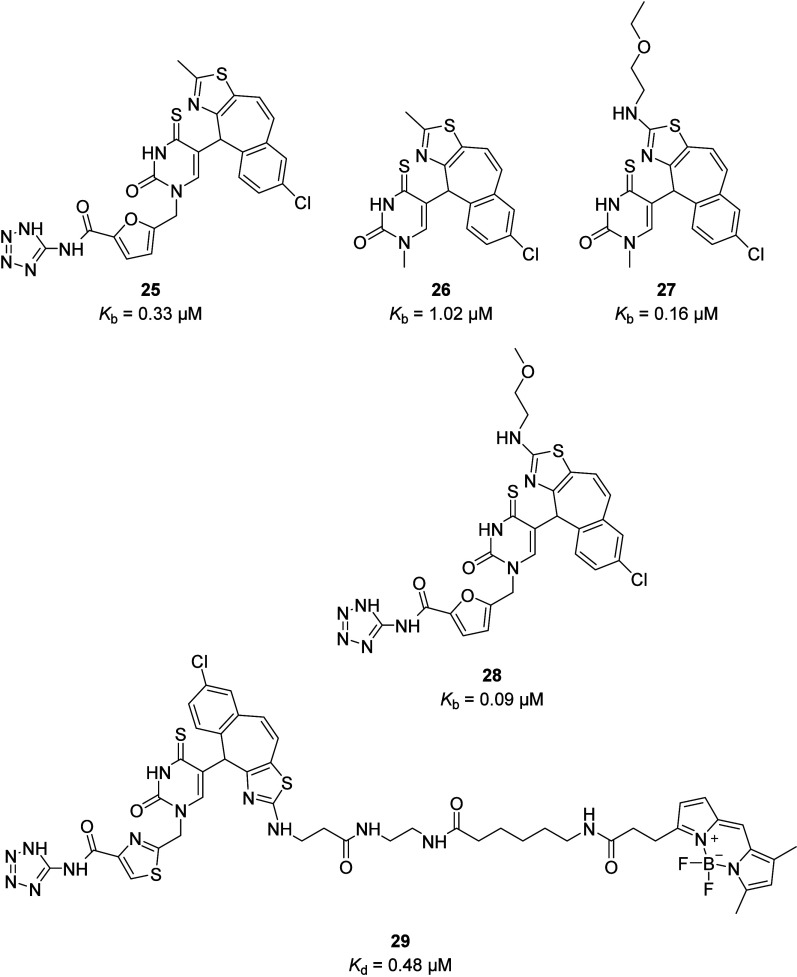
Novel antagonists of and the first fluorescent ligand for the P2Y_2_R, developed based on AR-C118925.

A new series of high-affinity P2Y_2_R antagonists with improved physicochemical properties were designed based on AR-C 118925 (**18**). The 2,8-dimethyl-5*H*-dibenzo[*a,d*][7]annulene tricycle of AR-C118925 (clogP = 5.3) was replaced with 7-chloro-4*H*-benzo[5,6]cyclohepta[1,2-*d*]thiazole to reduce the lipophilicity (**25**; clogP = 3.4 and *K*_b_ = 0.33 μM), and the acidic *N*-1 thiouracil substituent was removed to afford neutral P2Y_2_R antagonists (**26**; *K*_b_ = 1.02 μM), albeit with a reduction in potency. Potency was determined by using a fluorescence-based assay measuring the inhibition of UTP-induced intracellular Ca^2+^ release in P2Y_2_R-1321N1 cells. Activity was improved by replacing the thiazole 2-methyl substituent with linear, nonsterically demanding amino groups, such as **27** (*K*_b_ = 0.16 μM), and interestingly biological activity resided predominantly in the (*R*)-enantiomer. Reintroduction of the acidic *N*-1 thiouracil substituent, however, did not significantly increase the potency (**28**; *K*_b_ = 95.5 nM) despite its removal from AR-C 118925 abolishing activity (<50% inhibition at 10 μM), which could indicate a different binding mode for these antagonists. Nevertheless, the neutral P2Y_2_R antagonists retain comparable activity to AR-C 118925 while having improved physicochemical properties and therefore could have better oral bioavailability. Furthermore, the incorporation of a linker and fluorophore onto the thiazole produced the first fluorescent antagonists for the P2Y_2_R, such as **29** (*K*_d_ = 0.48 μM), allowing establishment of a new NanoBRET binding assay for the identification of P2Y_2_R fragments and ligands (Figure 11).^44^

**Figure 12:**
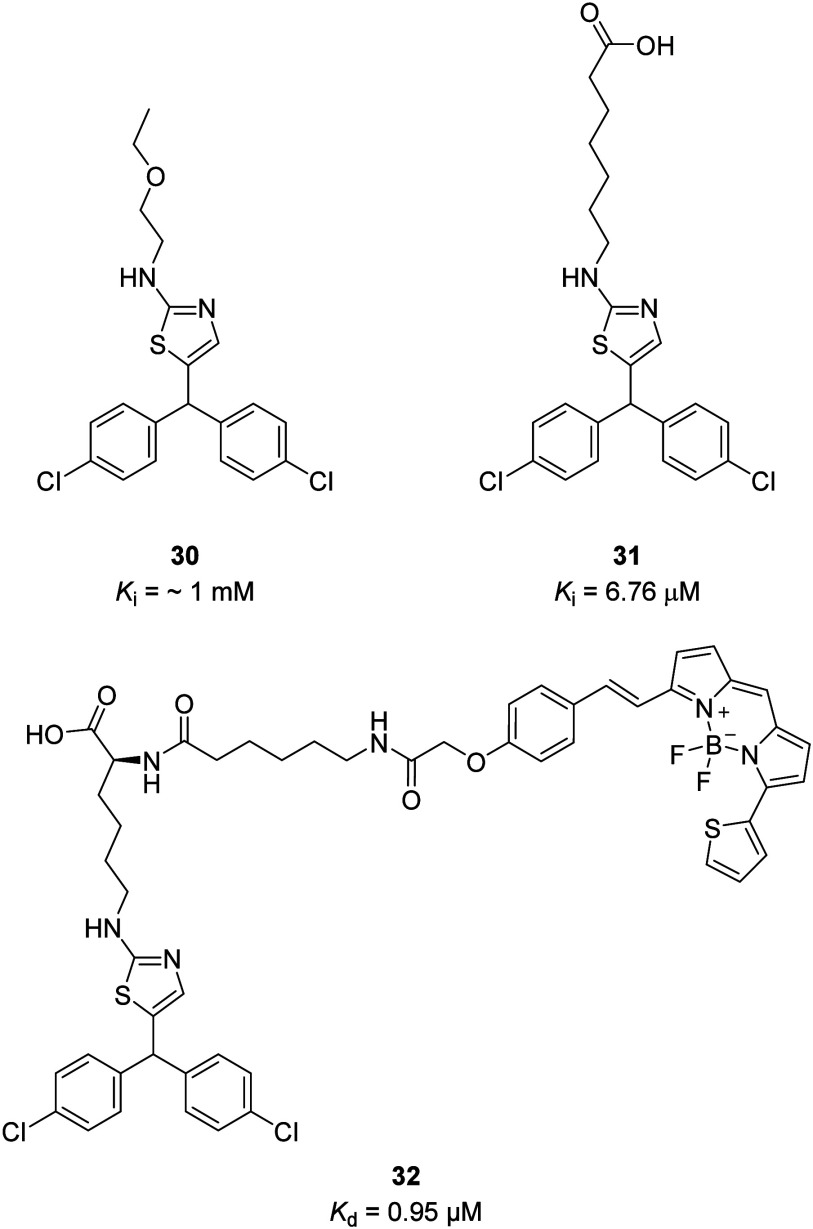
New chemotype P2Y_2_R antagonists with micromolar affinity and fluorescent ligands with distinctive binding.

New chemotype P2Y_2_R antagonists with micromolar affinity have been detailed based on **27** and its binding into the P2Y_2_R homology model, where it displayed a different predicted binding pose to AR-C 118925.^39,44,45^ Affinity was determined in NanoBRET competition binding assays with fluorescent ligand **29** in membrane preparations from 1321N1 astrocytoma cells stably transfected with P2Y_2_R tagged on the *N*-terminus with Nanoluciferase. Removing the seven-membered ring linking the tricycle and replacing the thiouracil with 4-chlorophenyl on **27** (*K*_b_ = 0.16 μM) significantly reduced the affinity (**30**; *K*_i_ = ∼1 mM). However, the affinity could then be improved at least 100-fold by incorporating terminal carboxylic acids onto the thiazole substituent (**31**; *K*_i_ = 6.76 μM) to promote proposed engagement with basic amino acids in the P2Y_2_R binding site. While displaying micromolar affinity, these compounds could offer starting points for further optimization by rational drug design to depart from AR-C118925 and its analogues. The new chemotype fluorescent ligand **32** (*K*_d_ = 0.95 μM) was designed based on these compounds and demonstrated distinct pharmacology compared to **29**, which suggests it occupies a different binding site on the P2Y_2_R and therefore presents new opportunities to target the receptor (Figure 12).^45^

## Antagonists of the P2Y_4_ Receptor

The P2Y_4_ receptor (P2Y_4_R) is a G_q_ protein-coupled receptor activated by UTP and is widely expressed in most human tissues. Inhibition of the P2Y_4_R has many potential clinical applications, including the treatment of cancer, pain, cardiovascular, and neurodegenerative disorders.^46^ However, no drug-like stable and selective P2Y_4_R antagonists have been disclosed to date.

**Figure 13:**
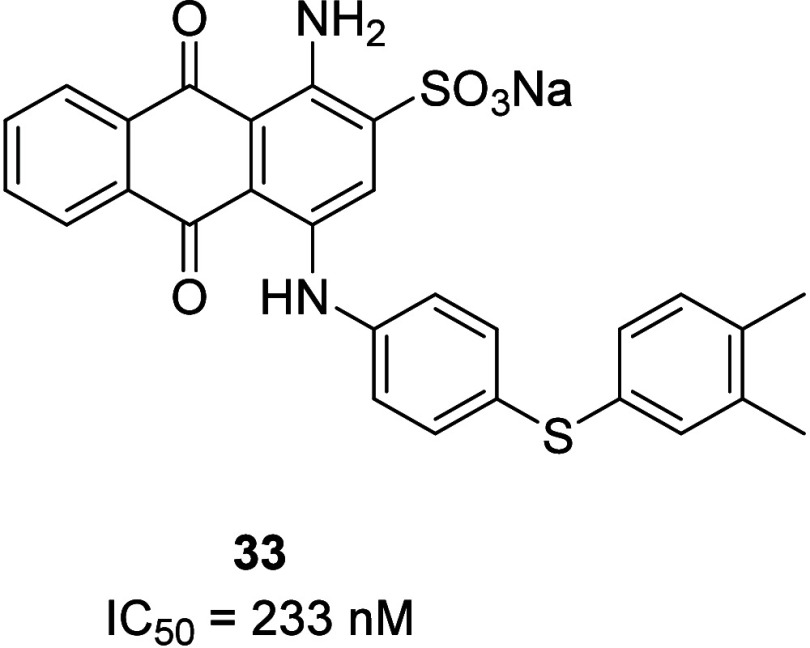
The most potent P2Y_4_ inhibitor based on an anthraquinone scaffold.

A series of sub-micromolar inhibitors based on an anthraquinone scaffold were recently disclosed.^47^ Potency was assessed by a fluorescence-based assay measuring the inhibition of UTP-induced intracellular Ca^2+^ release in 1321N1 astrocytoma cells stably transfected with the P2Y_4_R. The most potent compound **33** had an IC_50_ value of 233 nM and was shown to be selective against other P2Y receptor subtypes. Compound **33** is believed to act as an allosteric P2Y_4_R antagonist through evaluation of the concentration–response curves of UTP after preincubation with fixed concentrations of **33**. While the physicochemical properties would limit their use in *in vivo* applications, **33** could offer benefit as a pharmacological tool compound to study this interesting receptor (Figure 13).

## Antagonists of the P2Y_6_ Receptor

The P2Y_6_ receptor (P2Y_6_R) is a G_q_ protein-coupled receptor activated by UTP, which is widely expressed in various organs such as the brain, hypothalamus, hematopoietic cells, lymphocytes, alveoli, retina, colon, bladder, and peripheral blood. The P2Y_6_R engages in numerous physiological functions such as inflammation, energy metabolism, immune regulation, and cell proliferation. Inhibition of this receptor could therefore have clinical applications in various diseases, including cancer, hypertension, obesity, and diabetes, as well as bacterial infection.^48^

**Figure 14:**
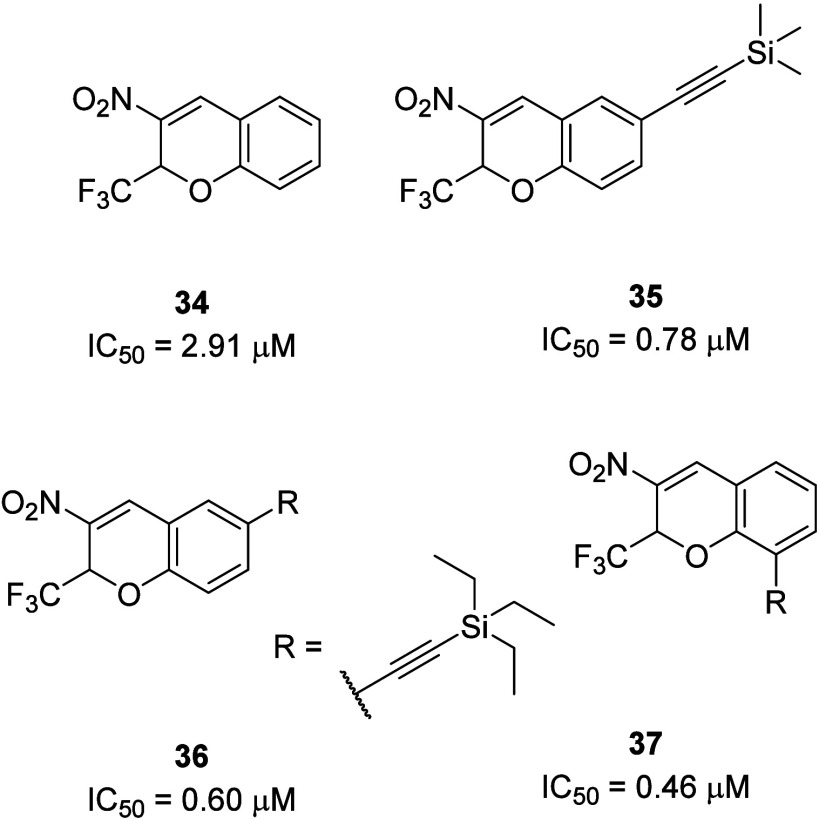
Chromene derivatives as surmountable P2Y_6_R antagonists.

Following the work in the synthesis of 3-nitro-2-(trifluoromethyl)-2*H*-chromene **34** by Ito, Jung reported further SAR studies on this P2Y_6_R antagonist series.^49,50^ Through evaluation of the concentration–response curves of UTP after preincubation with fixed concentrations of compound, **35** was demonstrated to be a surmountable P2Y_6_R antagonist (IC_50_ 0.785 μM) that was slightly more potent than the initial lead surmountable P2Y_6_R antagonist **34** (IC_50_ 2.9 μM) through the introduction of a trimethylsilyl acetylene moiety. In their follow-on work, they detailed further SAR studies and highlighted **36** (IC_50_ 0.604 μM) and **37** (IC_50_ 0.461 μM).^51^ It is likely their physicochemical properties would limit the use of these P2Y_6_R antagonists in *in vivo* disease models; however, they could offer benefits as pharmacological tool compounds to study this important receptor (Figure 14).

**Figure 15:**
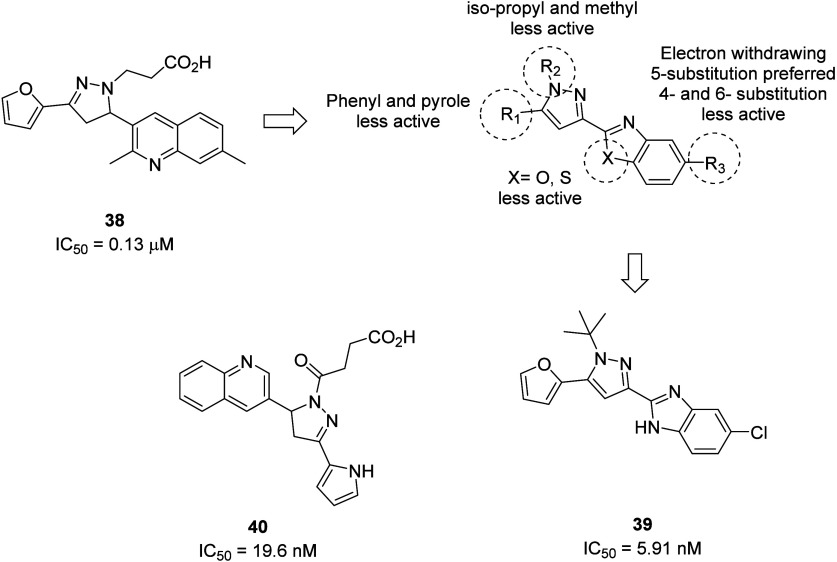
Novel pyrazole scaffold-based antagonists of the P2Y_6_R and a summary of structure activity studies around lead **39**.

A SBVS approach delivered the first drug-like small-molecule antagonists of the P2Y_6_R. A combination of molecular dynamics simulations and Molecular Mechanics/Generalized Born Surface Area (MM/GBSA) free energy calculations, followed by a classical SBVS pipeline, was employed using a P2Y_6_R homology model. P2Y_6_R antagonist activities from the *in silico* screening were evaluated in a HEK293 cell line expressing the P2Y_6_R. From the screening, initial hit compound **38** was found, which demonstrated activity in a concentration-dependent manner with an IC_50_ of 0.126 μM. Classical structure-based medicinal chemistry optimization resulted in **39** (IC_50_ 5.914 nM). A summary of the reported structure–activity relationship studies for this new chemotype is shown in Figure 15, highlighting the importance of the bulky *tert*-butyl R_2_ group; the furan R_1_ group and the optimal R_3_ chlorine atom were required for high P2Y_4_R activity. To demonstrate binding to recombinant P2Y_6_, a grating-coupled interferometry assay showed binding with a *K*_D_ of 3.47 μM, and further studies demonstrated high selectivity over the P2Y_1,2,4,12,14_ receptors.^52^

The PK properties of compound **39** were investigated in rats following intravenous (iv, 2.0 mg/kg) and oral (po, 20 mg/kg) administration (Table S2). Compound **39** showed modest bioavailability (∼16%) and *iv* half-life (*t*_1/2_ = 34.5 min), allowing *in vivo* studies where **39** showed efficacy in a DSS-induced ulcerative colitis model in mice after intrarectal injection. Compound **39** significantly inhibited NLRP3 inflammasome activation characterized by the down-regulated expressions of NLRP3, ASC, and caspase-1 in colon tissue. In addition, **39** was studied in an LPS-induced acute lung injury mouse model. Significantly reduced infiltration of inflammatory cells in the lung tissue was observed along with partial restoration of the structure of lung tissue. Compound **39** treatment in LPS-treated mice also decreased the expression of ALI-related cytokines, including tumor necrosis factor-α (TNF-α) and interleukin-1β (IL-1β), in the lung tissues and bronchoalveolar lavage fluid (BALF).^52^

Potent, selective, and *in vivo* efficacious quinoline-pyrazole scaffold-based P2Y_6_R antagonists were discovered through a cell-based assay (CHO-P2Y_6_R cells) of 300 compounds. The Ca^2+^ levels of UDP-stimulated P2Y_6_R in the presence of potential antagonists were measured via a fluorometric imaging plate rendering (FLIPR) assay. After further optimization of initial hits, the most potent compound **40** (IC_50_ = 19.6 nM) was identified. Compound **40** shares very close similarities in structure and shape to the previously reported virtual screening hit **38**. Both compounds share an *N*-linked carboxylic acid chain extending from a pyrazole core squeezed between two heterocyclic rings. Although not a neutral compound like **39**, compound **40** demonstrated promising anti-inflammatory and drug-like properties such as improved bioavailability (*F* = 43%), subtype selectivity, half-life (*t*_1/2_ = 5 h), and stability, thus warranting further studies (Table S2, Figure 15).^53^

The identification of metabolically stable small-molecule P2Y_6_R antagonists has allowed concept testing in mouse models following oral dosing, demonstrating that P2Y_6_R antagonists block the inflammatory cascade to relieve inflammatory diseases. Further studies confirming the orthosteric or allosteric binding modes of these P2Y_6_R antagonists would prove enlightening due to the observed difference between binding affinity (*K*_D_ = 3.47 μM) and Ca^2+^ mobilization (IC_50_ 5.914 nM) for **39**.

## Antagonists of the P2Y_11_ Receptor

**Figure 16:**
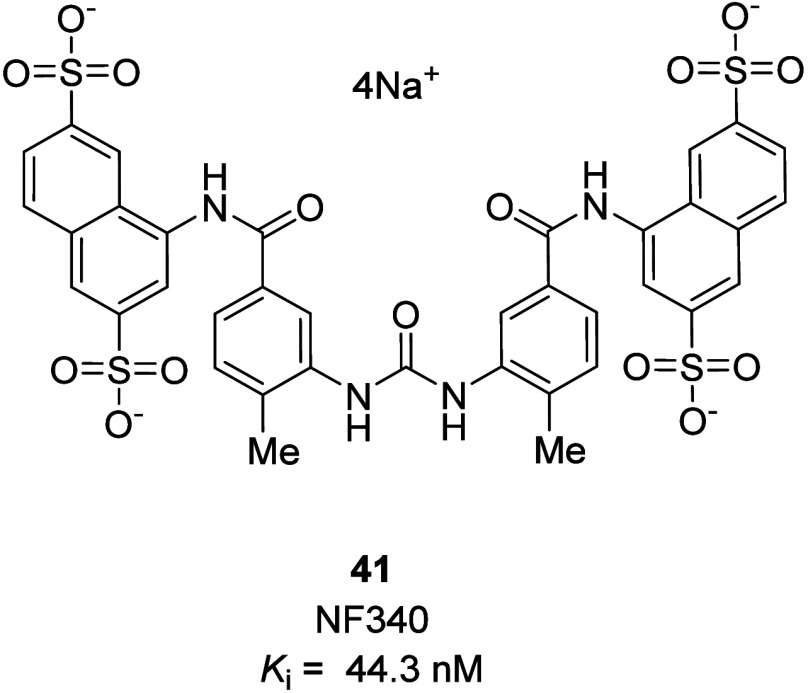
Chemical structure of NF340, a P2Y_11_R pharmacological tool.

The P2Y_11_ receptor (P2Y_11_R), primarily activated by ATP, is a unique receptor that couples to both G_q_ and G_s_ proteins. The G_s_ protein stimulates adenylyl cyclase, leading to the production of 3′,5′-cyclic monophosphate (cAMP), a secondary messenger for which downstream effects include the activation of cAMP-dependent protein kinase A (PKA). P2Y_11_Rs are expressed in various immune and blood cells involved in inflammatory responses, indicating their potential therapeutic applications in immunomodulation and inflammation. The absence of P2Y_11_Rs in rodents along with the limited availability of pharmacological tools has made studying their distribution and function more challenging.^54−57^ Currently, only a few antagonists are available for the P2Y_11_R, primarily suramin analogues like NF340 (41).^58^ There are no recent reports of novel drug-like antagonists for this receptor. Due to its high selectivity and potency, 4,4′-(carbonylbis(imino-3,1-(4-methyl-phenylene)carbonylimino))bis(naphthalene-2,6-disulfonic acid) tetrasodium salt (**41**, NF340) is considered a useful compound for determining the physiological function of the P2Y_11_R. However, the bulky and ionized nature of NF340 would limit the further progress of this antagonistic chemotype (Figure 16). However, it is still a valuable research tool. For instance, recent research using NF340 showed that antagonism of the P2Y_11_R has anti-inflammatory effects and potential in the treatment of conditions such as rheumatoid arthritis, atherosclerosis, and vascular inflammation.^59−61^ As our understanding of the involvement of the P2Y_11_R in various physiological and pathological processes grows, there is an increasing need for diverse, selective, and potent antagonists to explore receptor therapeutic potential and function. Also, the recent availability of X-ray crystal structures for the P2Y_1_ and P2Y_12_ receptors offers the opportunity for better prediction of P2Y_11_R tertiary structures along with the existing data.^62^ These efforts could potentially lead to new therapeutic strategies for inflammatory and immune-related disorders.

## Antagonists of the P2Y_12_ Receptor

The P2Y_12_ receptor (P2Y_12_R) plays a crucial role in platelet aggregation and thrombus formation, making it a key target for antithrombotic therapies. Identified in 2001, this G_i_-coupled GPCR is primarily expressed in platelets and responds to ADP as its endogenous agonist. The G_i_ protein inhibits adenylyl cyclase activity, leading to a reduction in the levels of the secondary messenger cAMP. While ADP activates both P2Y_1_ and P2Y_12_ receptors on platelets, the P2Y_12_R is particularly important for amplifying and sustaining the initial activation triggered by the P2Y_1_R. This amplification process involves decreased intracellular adenylate cyclase activity and prolonged calcium signaling, ultimately leading to enhanced platelet activation and stable thrombus formation.^63^ The P2Y_12_R’s specific expression pattern and central role in platelet function have made it an attractive target for antithrombotic drug development, thus reversible and irreversible inhibitors of the P2Y_12_R are on the market.^64−66^ P2Y_12_R antagonists have proven effective in preventing myocardial infarction and stroke.^67^ While the receptor’s function is best understood in platelets, ongoing research continues to uncover its importance in immune cells and the CNS. Recent studies have revealed potential application for antagonists in the treatment of inflammatory and neuropathic pain.^68^

**Figure 17:**
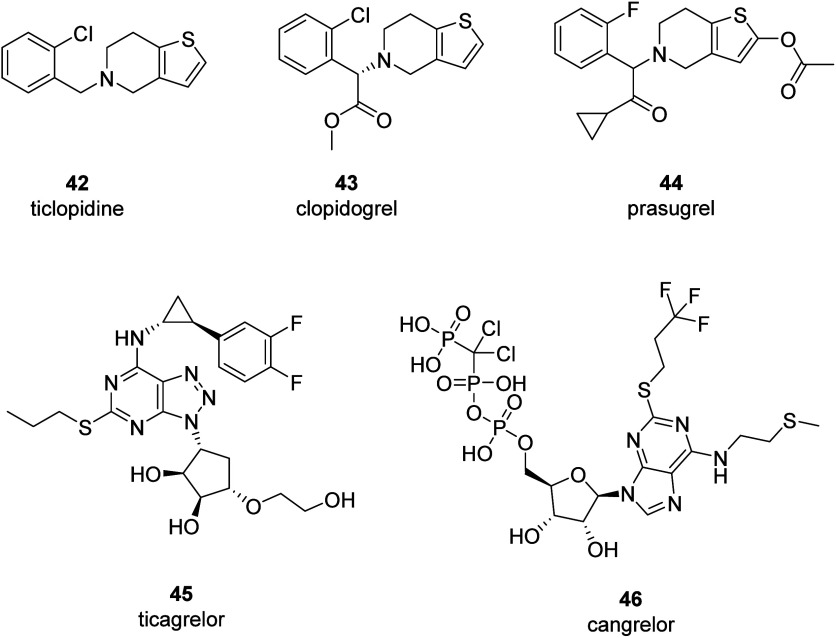
Structures and names of all commercial P2Y_12_R antagonists used as antithrombotic agents.

The first P2Y_12_R antagonist to be marketed was ticlopidine (**42**). However, it had serious hematological side effects like neutropenia and thrombocytopenia, as well as liver toxicity. Clopidogrel (**43**), launched in 1998, offered an improved safety profile and became a mainstay in treatment. Clopidogrel is a thienopyridine class antiplatelet agent that functions as an irreversible antagonist of the P2Y_12_R. It has been among the world’s best-selling drugs in recent years. However, as a prodrug clopidogrel requires hepatic bioactivation through a two-step process involving various CYP enzymes to generate its active metabolite.^69^ Prasugrel (**44**), which was launched in 2009, is the latest member of the thienopyridine class, with higher potency and a quicker onset of action than clopidogrel but with an increased bleeding risk.^70^ The reversible nucleoside/nucleotide analogues like ticagrelor (**45**) and cangrelor (**46**) represent significant advancements, offering potent platelet inhibition without requiring bioactivation. Ticagrelor, an orally bioavailable drug, demonstrates superior efficacy in acute coronary syndromes, while cangrelor, the only intravenous P2Y_12_R antagonist, is specifically approved for percutaneous coronary interventions due to its rapid onset (Figure 17).^71,72^

**Figure 18:**
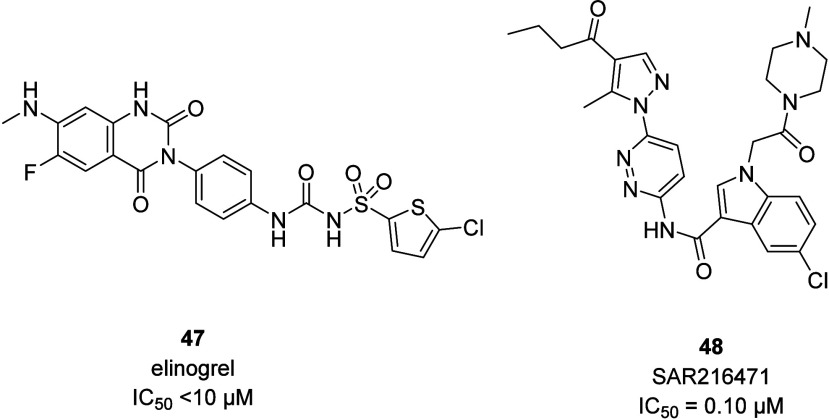
Chemical structure of clinical candidates elinogrel and SAR216471.

These P2Y_12_R antagonists, though widely used for the prevention of atherothrombotic events, are not without their limitations and potential side effects. Bleeding is the most common and serious side effect of all antithrombotics.^73^ Ticagrelor can cause dyspnea, especially in patients with underlying heart failure or pulmonary disease. This is often transient and can be managed by adjusting the dose or discontinuing the medication. Additionally, clopidogrel may be less effective in patients with certain genetic variations that affect its bioactivation. Considering these factors, there is a focus on the development of inhibitors that provide rapid, potent, and reversible platelet inhibition while offering an improved safety profile compared to existing agents. Industrial groups such as Novartis, Pfizer, and AstraZeneca have identified and optimized non-nucleotide small-molecule antagonists of the P2Y_12_R. For of the first candidates, elinogrel (**47**), whose clinical development was supported by Portola pharmaceuticals and then by Novartis, further progress was stopped in 2012.^66,74^ Similarly, there are no indications on further development of Sanofi’s clinical candidate SAR216471 (**48**).^75^ A recent study reported strong off-target activities for elinogrel, indicative of the increased bleeding risk associated with it (Figure 18).^76,77^

**Figure 19:**
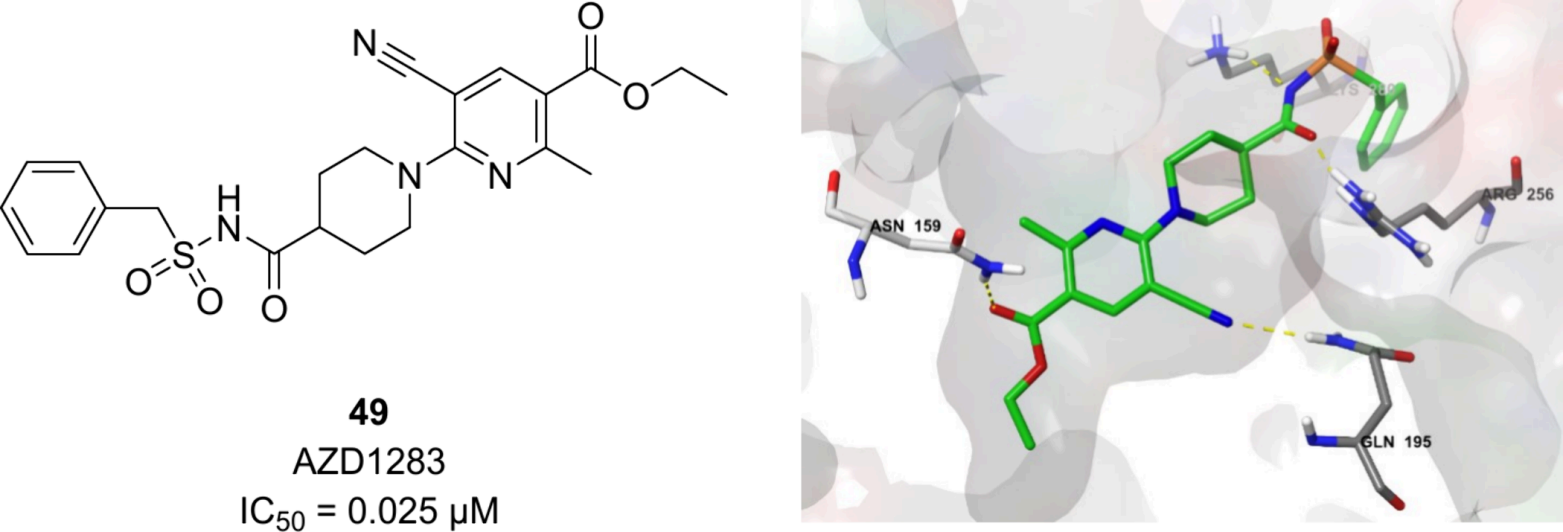
Chemical structure and binding pose of AZD1283 to the P2Y_12_ receptor (PDB ID: 4NTJ). Graphics generated using the PyMOL Molecular Graphics System, ver. 3.0, Schrödinger, LLC.

**Figure 20:**
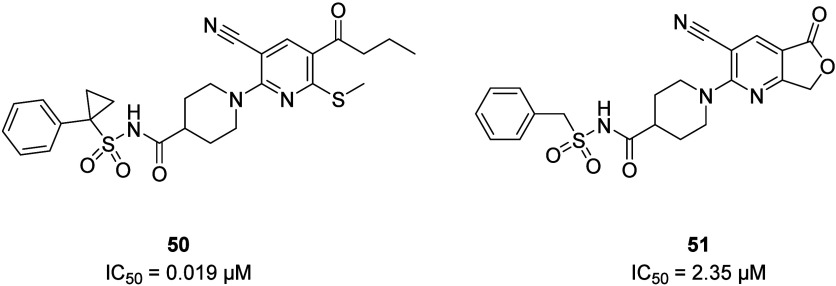
Metabolically stable analogues of AZD1283.

In the initial dose-escalating studies in humans, AstraZeneca’s clinical candidate AZD1283 (**49**) showed detectable activity only in very high doses due to low uptake and metabolic instability of the nicotinate ester. Modifying the ethyl ester to the corresponding propyl ketone, replacing the benzyl methylene group with a cyclopropyl group, and introducing methyl thioether in the pyridine 2-position gave the metabolically stable and highly active compound **50**. Unfortunately, the bleeding risk was high for this compound and therefore it was not progressed further.^66,78^ In the antagonist-bound crystal structure of the P2Y_12_R (PDB ID: 4NTJ), the ester carbonyl group of AZD1283 acts as a hydrogen bond acceptor for Asn159 and is believed to be crucial for its high affinity (Figure 19).^79^ Cyclization of this ester group to the *ortho*-methyl generated novel lactone analogues of AZD1283, often with good potency (*e.g.*, **51**, IC_50_ = 2.35 μM) and improved metabolic stability (Figure 20).^80^

**Figure 21:**
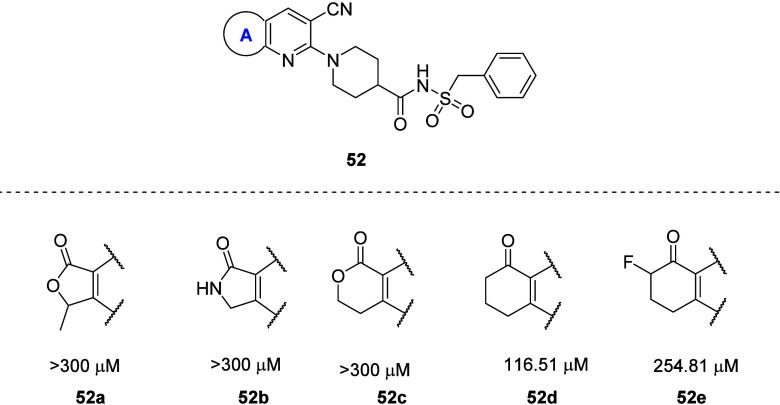
Exploration of the ring “A” SAR of 5-membered lactone ring-containing P2Y_12_R antagonists. The IC_50_ value measured by the platelet aggregation assay is provided for each compound.

Upon initial investigation of the ring “A” SAR by Kong et al., the five-membered lactone ring (**51**) showed significantly high activity in comparison to other cyclic systems such as lactams and cyclic ketones (**52a**–**e**) in the PRP aggregation assay (Figure 21). It is interesting to note that most of these analogues, if docked to the X-ray crystal structure of the P2Y_12_R, would have similar poses in the binding site, yet they show large discrepancies between potencies in the *in vitro* assay.^80^ With the current advent of artificial intelligence (AI) and its applications in drug discovery, such strict SAR results underline the importance of verifying the computational results experimentally before making key decisions in a drug discovery process.

**Figure 22:**
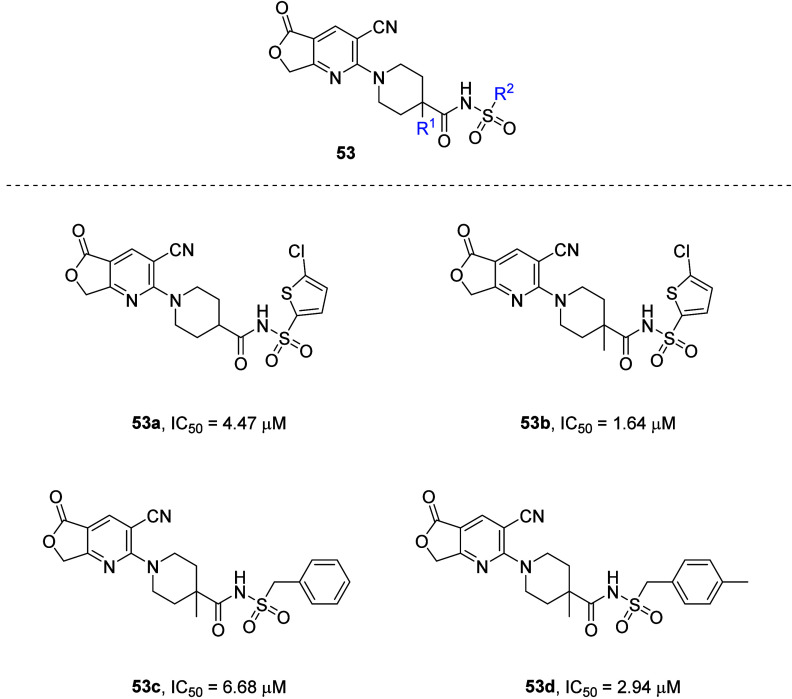
Selection of compounds synthesized for the evaluation of antagonistic potential at the P2Y_12_R.

To mask the metabolically labile positions on the five-membered cyclic lactone analogue **51** (IC_50_ = 2.35 μM), substitutions were introduced at the α-carbonyl position on the piperidine ring. This resulted in a loss of potency (**53c**; IC_50_ = 6.68 μM). Therefore, the 5-chlorothiophene fragment of elinogrel was tried as a replacement of the phenyl tail, which recovered the P2Y_12_R inhibitory potency (**53a** and **53b**; IC_50_ = 4.47 and 1.64 μM, respectively). However, the known off-target effects of elinogrel might have forced the team to reconsider the phenyl tail. In this regard, to increase the interactions of ligand with the hydrophobic pocket around the phenyl ring, small lipophilic groups were introduced on the *para*-position, which furnished the best-balanced compound in terms of potency and drug-like properties (**53d**; IC_50_ = 2.94 μM) (Figure 22).^80^

The PK properties of **53d** were assessed in Sprague–Dawley (SD) rats dosed orally at 5 mg/kg, and the compound demonstrated promising levels of high peak plasma concentration and exposure along with a long elimination half-life (*C*_max_ = 1661 ng/mL, AUC = 4120 ng·h/mL, *t*_1/2_ = 2.91 h). The overall profile of **53d** is comparable to that of AZD1283 in terms of CYP inhibition, and **53d** also demonstrated significantly better solubility. None of the compounds inhibited *h*ERG at concentrations as high as 40 μM. Significant antithrombotic effect was observed *in vivo* in a FeCl_3_-induced carotid artery thrombosis model with an oral dose of 10 mg/kg. Upon further testing, compound **53d** displayed dose-dependent inhibition of platelet aggregation, although at a higher dose (ED_50_ = 27 mg/kg) than that of clopidogrel. However, studies on bleeding time and bleeding weight after oral administration of clopidogrel and **53d** in a rat tail-bleeding model 1.5 h postdosing revealed a higher therapeutic window for compound **53d** than clopidogrel. Therefore, **53d** has potential applications in maintenance therapy where reduced bleeding risk is an important aspect.^80^

**Figure 23:**
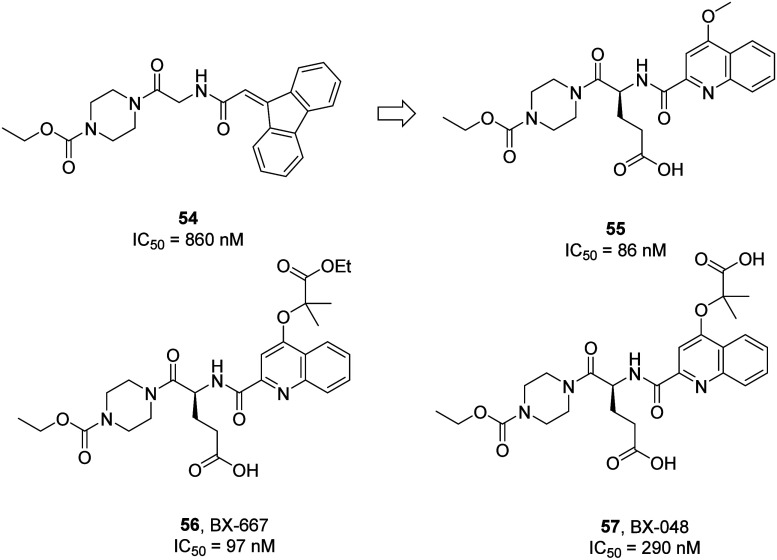
Discovery and optimization of clinical candidates BX-667 (**56**) and BX-048 (**57**), which target the P2Y_12_R.

Zetterberg and Svensson in 2016 noted, “unfortunately we are left a little in the dark with regards to Berlex discovery program leading up to the finding of BX667”.^66^ However, now the situation has changed. A recent report disclosed Berlex’s discovery of a fluorenylmethyl group containing piperazinyl carbamate as a P2Y_12_R inhibitor through a HTS program. Further optimization of the HTS hit **54** (IC_50_ = 860 nM) using parallel synthetic approaches resulted in the lead compound **55** (IC_50_ = 86 nM).^81^ Alterations to the ether functionality on the 4-position of the heterocyclic quinoline resulted in the clinical candidate BX-667 (**56**; IC_50_ = 97 nM) and its metabolite BX-048 (**57**; IC_50_ = 290 nM) with promising PK and pharmacodynamic (PD) properties (Figure 23).^82,83^ Following this, both Pfizer and Sanofi also disclosed piperazinyl carbamate compounds targeting the P2Y_12_R. However, no piperazinyl carbamate candidates are currently undergoing clinical studies to the best of our knowledge, except Actelion/Idorsia’s clinical candidate selatogrel (**58**, ACT-246475).^84−88^

**Figure 24:**
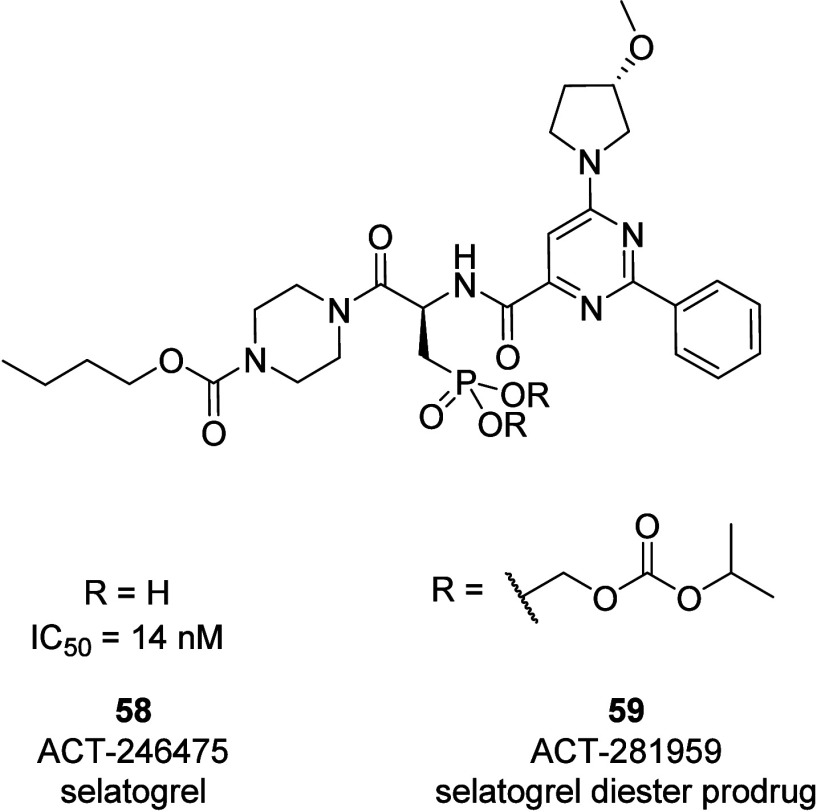
Chemical structure and other names of selatogrel (**58**) and its diester prodrug (**59**), which target the P2Y_12_R.

Selatogrel (**58**) is a potent, selective, fast-onset, and reversible inhibitor of ADP-induced platelet aggregation.^86,87^ This 2-phenylpyrimidine-4-carboxamide derivative binds to the P2Y_12_R with high affinity (*K*_d_ = 1.5 nM) and has an IC_50_ value of 14 nM in the platelet aggregation assay.^89^ Additionally, selatogrel has demonstrated equivalent antithrombotic efficacy and a better safety profile, with reduced bleeding risk and off-target effects in animal models.^86,90,91^ The first human trial of selatogrel and its diester prodrug ACT281959 (**59**) in 49 healthy males concluded that both the prodrug and the parent drug are well tolerated up to 1000 mg. However, the low systemic exposure of active drug was prohibitive for their further development as oral drugs.^88^ The phosphonate group of the parent drug selatogrel would be ionized at the physiological pH, which is not ideal for drug permeability after oral administration. On the other hand, the diester prodrug is a lipophilic high-molecular-weight (MW > 800, log *D* > 5.8) compound with limited aqueous solubility (Figure 24). In addition, the stepwise conversion of diester to the active drug is physiology-dependent. Considering these factors, it is not a surprise that subcutaneous administration rather than oral administration is proven to be effective in reducing platelet aggregation in acute coronary syndrome (ACS) patients. Results from the recently concluded phase 1 and phase 2 clinical trials warrant further studies of subcutaneously administered selatogrel in clinical scenarios.^92−95^ If successful, selatogrel could provide a quick onset/offset and potentially self-administrable antiplatelet agent, which would be beneficial in the prevention of thrombus development in the critical first few hours of a cardiac emergency.^96^

**Figure 25:**
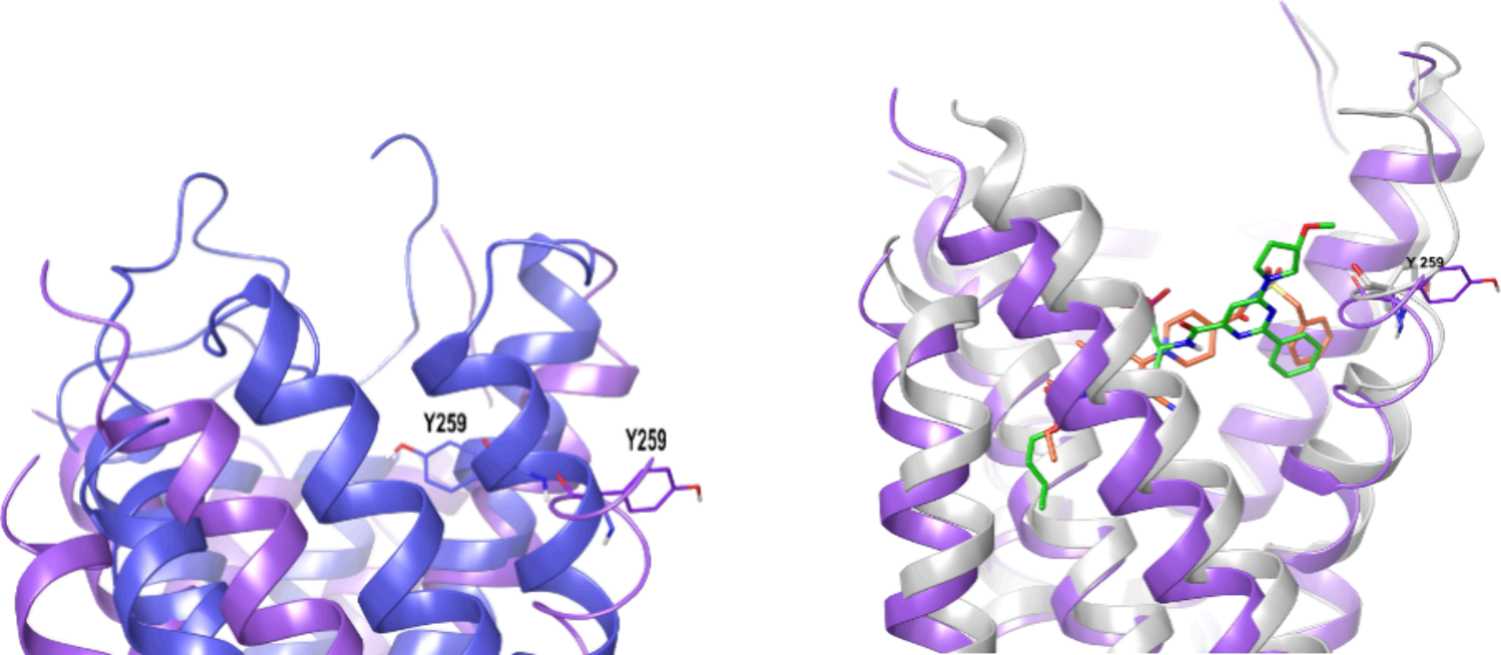
(Left) Overlay of agonist-bound (2-MeSADP, blue) and antagonist-bound (selatogrel, grape) X-ray crystal structures showing a tight structure for the P2Y_12_R after agonist binding; a representative residue Tyr259 is shown as sticks to demonstrate the inward shift after agonist binding (on the left). (Right) Overlay of two antagonist-bound structures showing similarities in receptor conformation in the inactive state (on the right). AZD1283 (49, as orange core sticks) is bound to the P2Y_12_R (white ribbon), and selatogrel (**58**, green carbon sticks) is bound to the P2Y_12_R (grape ribbon). Graphics using the PyMOL Molecular Graphics System, ver. 3.0, Schrödinger, LLC.

Agonist-bound X-ray cocrystal structures of the P2Y_12_R (PDB IDs: 4PXZ, 4PY0) showed significant conformational differences compared to that of the antagonist bound inactive conformation (PDB ID: 4NTJ). The binding of potent agonist 2-MeSADP induced conformational changes in an unprecedented manner, resulting in a tight structure of the receptor.^97^ The extracellular binding pocket of the P2Y_12_R showed high plasticity and striking differences in the agonist- and antagonist-bound structures. As in the case of the P2Y_1_R, previous mutagenesis and modeling studies could identify key residues such as Arg256, Lys280, and Tyr259 that are involved in the agonist binding.^98−100^ However, the agonist-bound experimental structure sheds further light on the roles of other residues such as Arg93 and Lys174. The availability of two highly divergent 3D structures of the P2Y_12_R will encourage the structure-based drug design of novel antagonists with better properties.^101^ The selatogrel-bound cocrystal structure of the P2Y_12_R (PDB ID: 7PP1) resembled the AZD1283-bound inactive state but with Tyr259 pushed further out of the binding pocket (Figure 25).^76^ Moreover, selatogrel behaved as an inverse agonist of ADP-independent constitutive P2Y_12_R signaling, both in a recombinant cellular system and in human platelets. Selatogrel acted as a potent inverse agonist of the P2Y_12_R, blocking the basal receptor-dependent G_i_ signaling, as confirmed by an increase in basal cAMP levels. Constitutive signaling of P2Y_12_R is particularly important in diabetic patients. They are reported to have a raised prothrombotic profile due to higher P2Y_12_R expression and platelet activity. Therefore, using an inverse agonist to block the binding of the orthosteric ligand ADP and to switch the receptor from an active to an inactive conformation would be beneficial in diabetic patients.^76^

**Figure 26:**
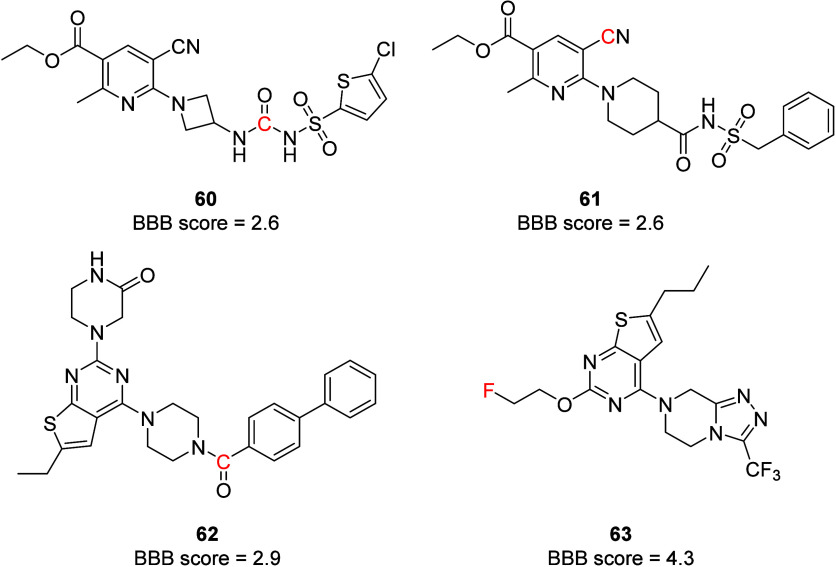
P2Y_12_R-based PET tracers. Atoms shown in red indicate the position of radiolabeling. ^11^C or ^18^F isotopes were used.

The P2Y_12_R is also a promising target for understanding CNS diseases because of their distribution in microglia.^102^ Microglia are the immune cells of the CNS and they are the brain’s first responders to injury and neurodegenerative diseases. PET tracers targeting the P2Y_12_R with good brain penetrability could be valuable tool compounds for studying CNS disorders like Alzheimer’s disease, multiple sclerosis, and Parkinson’s disease.^103,104^ Unfortunately, most of the non-nucleotide P2Y_12_R antagonists have high molecular weights (>500 Da) and many H-bond donor and H-bond acceptor groups, making topological polar surface area (TPSA) scores >125 Å. Other issues include the presence of highly polar groups, low-p*K*_a_ functionalities, and multiple aromatic rings affecting their blood–brain barrier permeability (BBB score <4).^105^ Thienopyridine and similar analogues have more appropriate physicochemical properties for targeting the CNS, but their slow onset of action and irreversible binding characteristics are not ideal for CNS PET tracing applications. Because of these issues, attempts to develop P2Y_12_R PET tracers (**60**–**63**, Figure 26) so far have encountered challenges such as poor CNS uptake (**60** and **62**) and P-glycoprotein (P-gp) efflux (^18^F-thienopyrimidine **63**).^106−108^ Ma et al. opined that the ADP binding pocket in the P2Y_12_R might be a poor choice to develop brain-penetrant PET tracers because the ligand binding in this site requires multiple hydrogen bonds and other interactions, as in the case of 2-MeSADP.^68^ Structure-based approaches to design compounds mimicking non-nucleotide antagonist binding could provide compounds with a good BBB score, as this pose relies more on π–π stacking and other hydrophobic interactions.

Pharmaceutical companies have shown special interest in the P2Y_12_R subtype compared to other purinergic transmembrane receptors. This can be attributed to their exclusive distribution in platelets and brain tissue. The commercial success of marketed drugs such as clopidogrel, prasugrel, and ticagrelor have encouraged further research in this area, which has led to the discovery of multiple oral small-molecule clinical candidates. Most of them have not made it into the clinic yet. However, the medicinal chemistry approaches employed to address the unique challenges in the development of non-nucleotide antagonists of the P2Y_12_R have enriched lead optimization strategies in medicinal chemistry. Whether phase 2 clinical trial results of subcutaneous selatogrel will prompt others (like Pfizer, Sanofi, and Berlex) to revisit their piperazinyl carbamate candidates is yet to be seen. With the ever-increasing demand to study and treat CNS conditions, P2Y_12_R expression in the microglia also makes it an attractive CNS target. In this regard, the current situation demands CNS appropriate receptor antagonist development. Moreover, the radioligands used in binding assays are challenging to synthesize; therefore, development of tools for fluorescence-based binding assays such as bioluminescence resonance energy transfer (BRET) would be appealing.

## Antagonists of the P2Y_13_ Receptor

**Figure 27:**
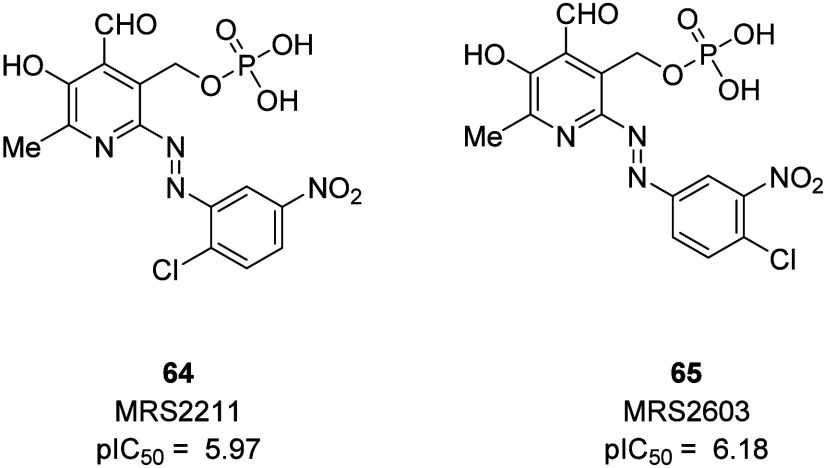
PPADS analogues as P2Y_13_ inhibitors.

Along with the P2Y_12_ and P2Y_14_ receptors, the P2Y_13_ receptor (P2Y_13_R) is a member of the G_i_-coupled P2Y receptor subgroup. It is primarily activated by ADP, although ATP is a partial agonist of this receptor. Given its roles in metabolism, bone homeostasis, and neuroprotection, the P2Y_13_R is being investigated as a potential target for various conditions.^109,110^ However, medicinal chemistry programs targeting P2Y_13_R are rare. The P2Y_12_R antagonist cangrelor has been shown to also inhibit the P2Y_13_R-regulated capacity of megakaryocytes to produce pro-platelets. Ticagrelor also acted as a P2Y_13_R antagonist *in vitro* but did not share the functional effects of cangrelor.^111^ The subtype-selective antagonist tool compounds MRS2211 and MRS2603 were developed for the P2Y_13_R based on the structure of PPADS (pyridoxal-5′-phosphate-6-azo-phenyl-2,4-disulfonate). PPADS is a P2 receptor antagonist with more than 10-fold selectivity for P2X over P2Y receptors. It is also a low potency antagonist for the P2Y_1_ and P2Y_13_ receptors with an IC_50_ of around 10 μM. The 2-chloro-5-nitro analogue (**64**, MRS2211, IC_50_ = 1.1 μM) and 4-chloro-3-nitro analogue (**65**, MRS2603, IC_50_ = 0.66 μM) of PPADS inhibited ADP-induced IP_3_ formation. Both analogues show selectivity for the P2Y_13_R over the P2Y_12_R despite these receptors being within the same P2Y receptor subgroup. MRS2211 also showed >20-fold selectivity for the P2Y_13_R over the P2Y_1_R, another ADP binding receptor.^112^ While these compounds are valuable research tools to study this important receptor, further progression is not warranted (Figure 27). Currently, there are no known specific drug-like antagonists for the P2Y_13_R. The lack of suitable ligands limits the ability to study P2Y_13_R function *in vivo* and to explore its potential as a therapeutic target. Therefore, the development of more selective and drug-like P2Y_13_R antagonists is highly desired.

## Antagonists of the P2Y_14_ Receptor

Unlike other P2Y receptors that are typically activated by ATP, ADP, or UTP, the P2Y_14_ receptor (P2Y_14_R) is primarily activated by extracellular UDP-sugars such as UDP-glucose and UDP-galactose. UDP-glucose is a potent agonist of P2Y_14_R, with studies showing EC_50_ values of around 40–80 nM. These receptors are implicated in various physiological processes, including immune responses, inflammation, and metabolic regulation. Their expression in immune cells, epithelial tissues, and the CNS highlights their broad physiological relevance, from mediating acute inflammatory responses in conditions like gout to contributing to chronic diseases such as diabetes, neuroinflammatory disorders, and cancer. Because of its pharmacological importance, P2Y_14_R antagonist development has attracted the particular interest of several academic research groups in recent years.^113−115^

**Figure 28:**
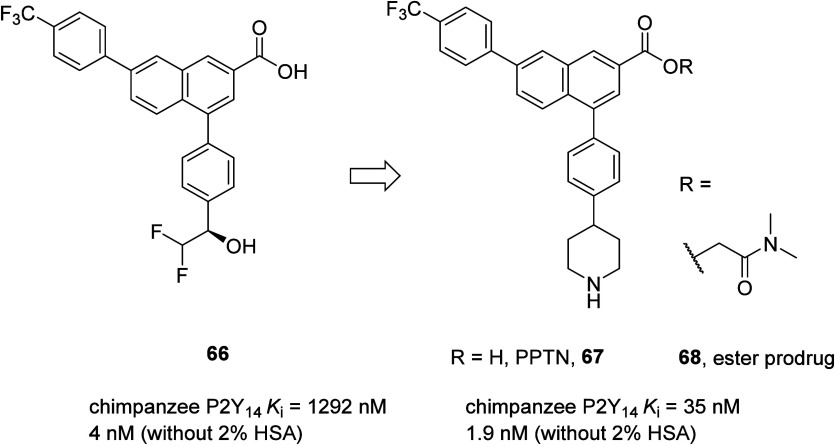
Optimization of the HTS hit **66** to high-affinity selective P2Y_14_R antagonist PPTN (**67**) and prodrug **68**.

The first drug-like antagonists of the P2Y_14_R were reported by an industrial research group. A HTS of the Merck-Frosst compound library, using a FLIPR Ca^2+^ mobilization assay in HEK293 cells and the following optimization of hits, identified the first non-nucleotide competitive antagonist of the P2Y_14_R (**66**).^116^ Efforts to decrease the high plasma protein binding (>99%) of this compound resulted in the discovery of zwitterionic 4-[4-(4-piperidinyl)phenyl]-7-[4-(trifluoromethyl)phenyl]-2-naphthalenecarboxylic acid (**67**, PPTN), with high affinity and selectivity toward the P2Y_14_R (Figure 28).^117^ Due to the poor bioavailability of PPTN (67) (5% in mice, 50 mg/kg po) and low aqueous solubility (<5 μg/mL), most of the recent lead optimization efforts are aimed at improving the physicochemical properties of PPTN. In the PK studies, the ester prodrug (**68**) afforded plasma levels of PPTN that were substantially higher than direct administration of the parent compound. However, to the best of our knowledge, data about the further progress of **68** have not been disclosed.

**Figure 29:**
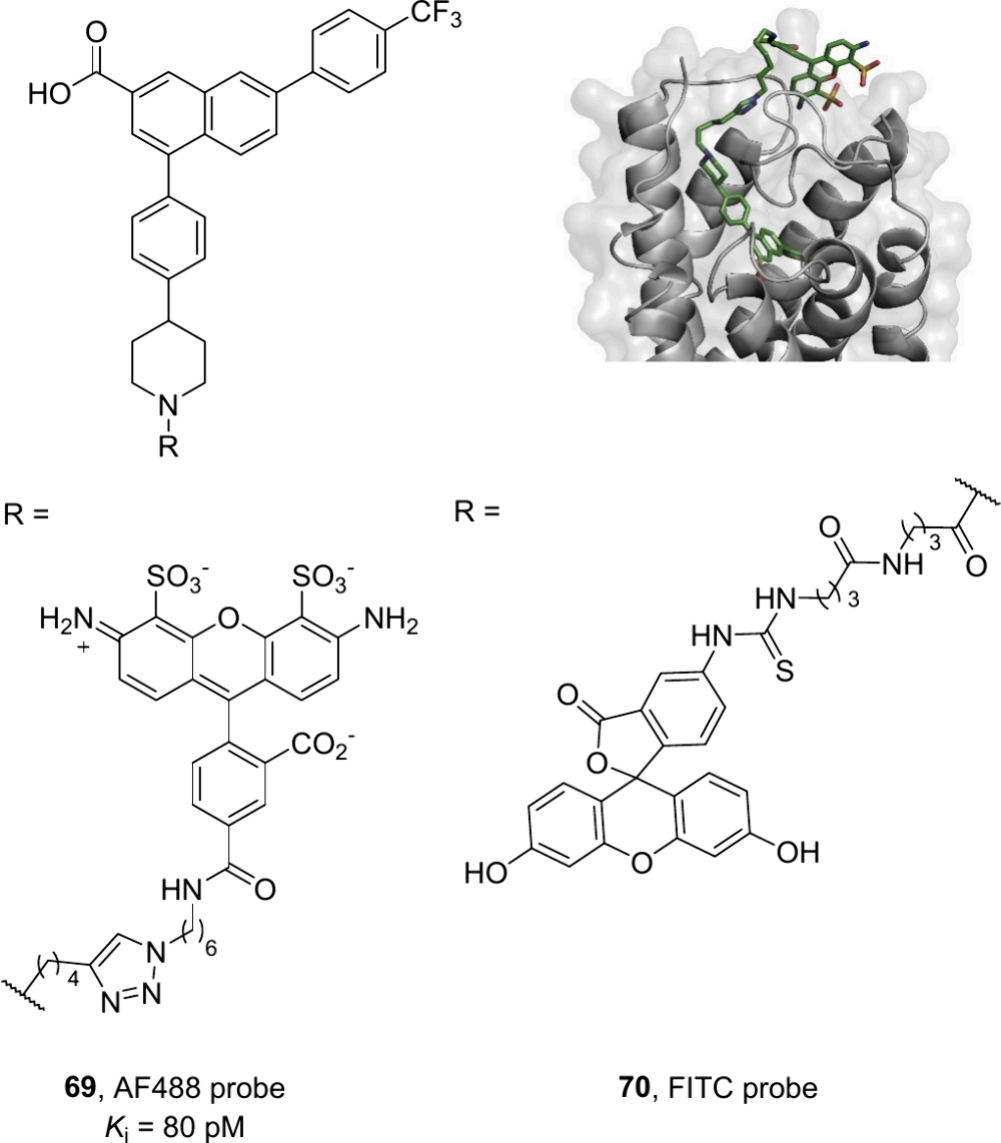
Fluorescent probes designed and synthesized for use in flow cytometry assays to profile P2Y_14_R antagonists. Binding of the AF488 probe (**69**) to the homology model of P2Y_14_ is shown in the top right. Graphics generated using the PyMOL Molecular Graphics System, ver. 3.0, Schrödinger, LLC.

The Jacobson group constructed homology models of the P2Y_14_R based on high-resolution X-ray crystal structures of the P2Y_12_R, which suggested that the piperidine ring is suitable for fluorophore conjugation while preserving affinity.^79,97^ Using this information, the Alexa Fluor 488 (AF488) containing ligand **69** (MRS4174, *K*_i_ = 80 pM) was identified as a high-affinity P2Y_14_R fluorescent probe with low nonspecific binding.^118^ Later, Wang et al. also reported an easy to make tracer with FITC as the fluorescent tag connected to PPTN via two molecules of 4-aminobutyric acid (**70**) to use as a tracer in the flow cytometric analysis of antagonists at the P2Y_14_R expressed in HEK293 cells (Figure 29).^119^

Isosteric replacement of the naphthalene rings with phenyl triazole and an amido phenyl ring was explored to optimize the physicochemical properties of PPTN (**67**).^120,121^ Structure-based design using the previously constructed homology model of the P2Y_14_R identified triazole-containing antagonist compound **71** with an IC_50_ value of 31.7 nM in a flow cytometry competition binding assay in P2Y_14_R-CHO cells. The antagonistic activity was further confirmed by cAMP measurements of P2Y_14_R expressed in CHO cells in the presence of compound **71**. Selectivity of **71** against other P2Y receptor subtypes such as P2Y_1,2,4,6,11_ in concentrations up to 10 μM was also demonstrated. The potencies of triazole analogues tested in this study were lower than that of PPTN (IC_50_ = 6 nM), and **71** also showed weak off-target interactions against other GPCRs. Additionally, the physicochemical properties of **71** have not been determined. The triazole core is expected to improve the solubility and could serve as a replacement for the hydrophobic naphthalene core of PPTN (**67**). The 3-amido benzoic acid analogue **72** (IC_50_ = 1.7 nM) showed comparable P2Y_14_R antagonist activity to PPTN (IC_50_ = 1.98 nM) when tested in a functional assay of the agonist-induced inhibition of cAMP production in the presence of 30 μM forskolin in THP-1 cells stably expressing the P2Y_14_R. Potent anti-inflammatory effects of **72***in vitro* were also demonstrated by measuring cAMP levels in monosodium urate (MSU) treated THP-1 cells. This series of 3-amido benzoic acid analogues of PPTN showed improved aqueous solubility and good microsomal stability.^120^

**Figure 30:**
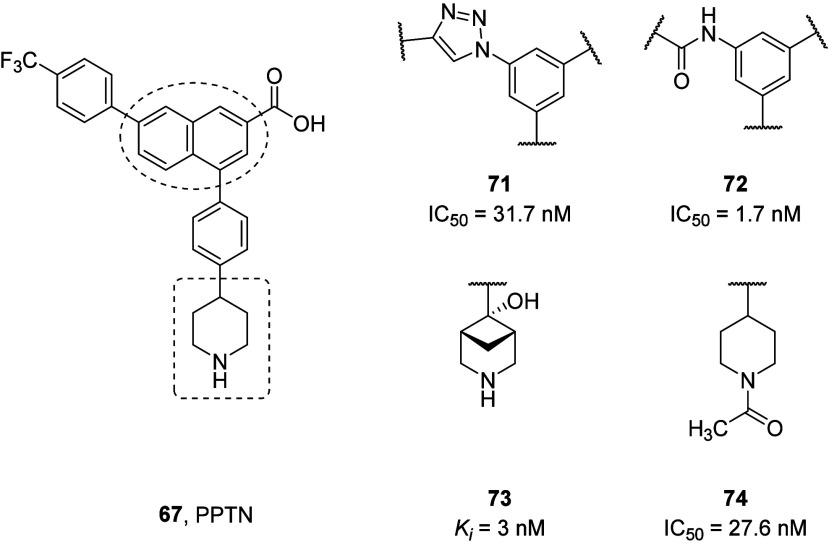
PPTN analogues as P2Y_14_R antagonists.

Efforts to modify the phenyl-piperidine part of PPTN (**67**) and its isosteric analogues revealed good affinity antagonists possessing heteroaromatic and heteroalicyclic substituents in place of the piperidine moiety.^122,123^ Constraining the piperidine ring of PPTN was found to be a productive means of increasing three-dimensionality and at the same time preserving or enhancing binding affinity.^124,125^ A potent, sterically constrained zwitterionic compound **73** (P2Y_14_R *K*_i_ = 3 nM) displayed desirable absorption, distribution, metabolism, excretion, and toxicity (ADMET) properties in both *in vitro* and *in vivo* studies. Furthermore, only weak *h*ERG inhibition with an IC_50_ > 30 μM was observed in addition to the absence of any major CYP inhibition. Compound **73** was highly efficacious upon oral administration (10 μmol/kg) and reversed chronic neuropathic pain in a mouse model (chronic constriction injury, CCI model). Additionally, **73** achieved full reversal of mechano-allodynia when administered as oral gavage in the CCI model with longer duration than PPTN. Favorable *in vivo* activity of **73** was also evidenced by reduced airway eosinophilia in a protease-mediated mouse model of allergic asthma. The oral activity of this compound *in vivo* suggests a possible H-bond between the “OH” group and “*N*” atom of the bridged piperidine, which would effectively reduce the zwitterionic character.^126^ Intramolecular H-bonds have been reported to increase the permeability, including oral bioavailability, of polar compounds and are used in the lead optimization stage of medicinal chemistry campaigns. The P2Y_14_R is a target for chronic neuropathic pain treatment. In this regard, *N*-acetyl derivative **74** was efficacious *in vivo* in reversing neuropathic pain resulting from peripheral nerve injury (Figure 30).^127^

**Figure 31:**
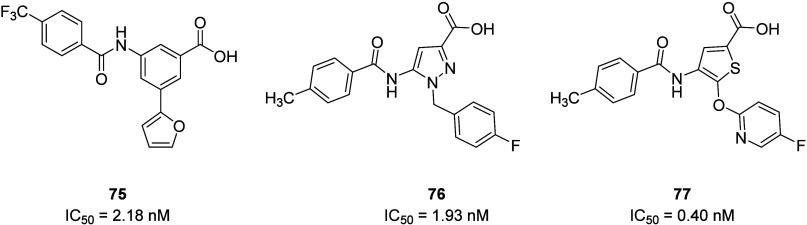
Novel P2Y_14_R inhibitors generated via bioisoteric and scaffold hopping strategies.

An amido-phenyl bioisostere strategy combined with the heteroaromatic replacement of the basic piperidine ring identified the furan-containing compound **75** with an IC_50_ value of 2.18 nM. Compound **75** showed improved bioavailability (*F* = 48%), good metabolic stability (*t*_1/2_ > 60 min, human microsomes), and increased water solubility (53 μg/mL) in comparison to PPTN (*F* = 7%, aq. solubility <5 μg/mL). *In vivo* anti-inflammatory evaluation of **75** in an MSU-induced mouse model demonstrated efficacy in alleviating mice paw swelling and inflammatory infiltration, although with a shorter duration than PPTN.^128^ However, this study demonstrated that the basic amino group is not mandatory for activity and provides an opportunity for further exploration of the SAR surrounding the piperidine ring of **67**. Scaffold hopping strategies to replace the naphthalene moiety with five-membered rings such as pyrazole and thiophene have also been successfully employed to address the physicochemical issues of existing P2Y_14_R antagonists. The exploration of the heterocyclic SAR led to the identification of compound **76**, a novel and potent P2Y_14_R receptor antagonist. This pyrazole derivative exhibited high binding affinity (IC_50_ = 1.93 nM) to the P2Y_14_R when tested in a functional assay measuring the antagonism of agonist-induced inhibition of cAMP production in the presence of forskolin (30 mM) in a P2Y_14_R-HEK293 cell line. Compound **76** displayed high selectivity for the P2Y_14_R (vs P2Y_1,2,4,6,12_), improved solubility (318.7 μg/mL at pH 7.4, clogP = 3.01) across various pH ranges relative to PPTN, and favorable PK properties, including shorter onset time, excellent microsomal stability both *in vitro* and *in vivo*, and moderate bioavailability (*F* = 43%) in rats. *In vitro* studies revealed extremely low cytotoxicity and significant anti-inflammatory effects. Moreover, compound **76** demonstrated substantial efficacy in a lipopolysaccharide (LPS)-induced acute peritonitis model, comparable to dexamethasone, as they both effectively reduced the levels of inflammatory factors in the peritoneal fluid of mice stimulated by LPS 6 h before fluid collection. Following the pyrazole series, 4-amide-thiophene-2-carboxyl derivatives were identified as novel potent P2Y_14_R antagonists. The optimized compound **77** exhibited subnanomolar antagonistic activity (IC_50_ = 0.40 nM) and remarkable *in vivo* efficacy in a dextran sodium sulfate (DSS)-induced colitis mouse model. These findings underline that P2Y_14_R antagonists with favorable druggability profiles are potential candidates for the treatment of various inflammatory diseases (Figure 31).^119,129^

**Figure 32:**
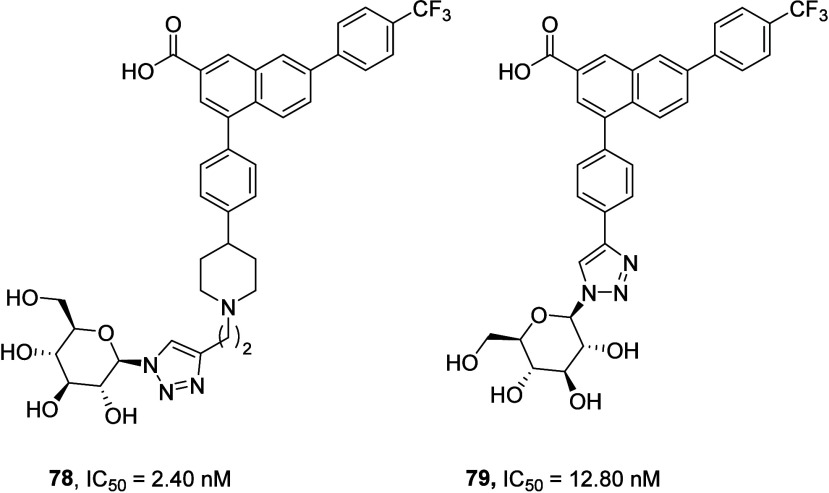
Glycoconjugates of PPTN as novel P2Y_14_R inhibitors.

The latest members of the PPTN based P2Y_14_R antagonists are glycoconjugates of PPTN designed to exploit the glucose binding site of UDP-glucose on the P2Y_14_R. Both triazolyl *N*-linked glucose conjugate **78** (MRS4865, IC_50_ = 2.40 nM) and carbohydrate conjugate **79** (IC_50_ = 12.80 nM) with a triazole spacer instead of the piperidine ring showed promising *in vivo* results in animal models of inflammatory conditions such as asthma and neuropathic pain. *In vitro* affinities were measured by binding assays using fluorescent tracer **69** in P2Y_14_R-expressing CHO cells. Detailed PK and PD study data have not been disclosed; however, enhanced aqueous solubility compared to PPTN has been reported (Figure 32).^130^

**Figure 33:**
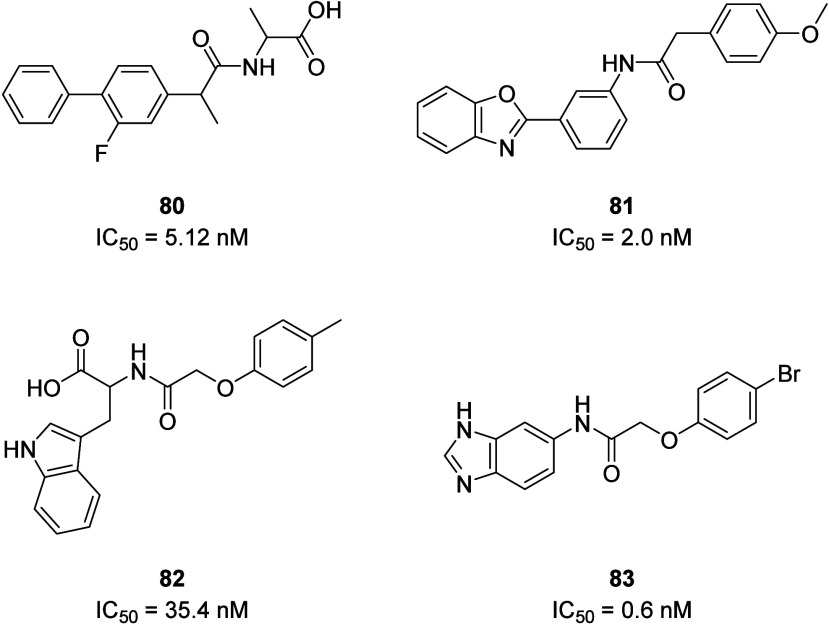
P2Y_14_R antagonists discovered via SBVS and optimization.

SBVS has been widely used in lead compound identification as a complementary strategy to HTS. Even though the crystal structure of the P2Y_14_R has not yet been reported, Li and co-workers used well-established homology models of the P2Y_14_R to virtually screen a commercial library. The homology models were previously developed by Jacobson and co-workers based on the agonist-bound P2Y_12_R receptor structure and were validated using known ligand structure–activity relationship (SAR) data.^131−133^ These P2Y_14_R homology models were selected, optimized, and applied in a Glide docking-based virtual screening (VS) campaign. The SBVS identified compounds with potent P2Y_14_R binding affinity and nanomolar *in vitro* activity, many of which had a carboxylic acid functionality linked to an aryl ring by various linker moieties (*e.g*., compound 80). However, it is interesting that PPTN (**67**) could not produce acceptable docking poses and may have adopted a distinct binding mode compared with the hit compounds of this work. In a follow-up work, by comparing the binding poses of these hit molecules using MM/GBSA binding free energy calculations and decompositions, two almost symmetrical ligand binding pockets were identified. Rational compound design targeting the residues in these two pockets followed by chemical synthesis and biological evaluation resulted in neutral drug-like 2-phenyl-benzoxazole acetamide derivative **81** (Figure 33). Compound **81** displayed potent P2Y_14_R antagonistic activity (IC_50_ = 2 nM) and *in vivo* potency in a mouse acute gout model.^131,134^

More *N*-heterocyclic acetamide derivatives as neutral drug-like P2Y_14_R antagonists were discovered via rational compound design based on previous hit compounds. Crystallographic overlays of **81** and **82** suggested the carboxylic acid functionality on **82** almost had no beneficial interaction with the surrounding residues in the P2Y_14_R homology model. Therefore, a library of hybrid compounds containing fragments similar to those of **81** and **82** were designed and synthesized. The quick and straightforward synthesis of **81** and **82** hybrids allowed rapid *in vitro* screening of a small compound library. Briefly, different commercially available arylamines were reacted with 2-(4-substituted)phenoxy acetic acids via classical HATU coupling reactions to access ∼25 analogues. The hybrid analogues displayed highly divergent SARs. Benzimidazole and quinoline rings (**83–88**) were found to be suitable heterocyclic fragments with antagonistic potencies ranging from 0.6 to >35 nM (Table 1).^135^

**Table 1:**
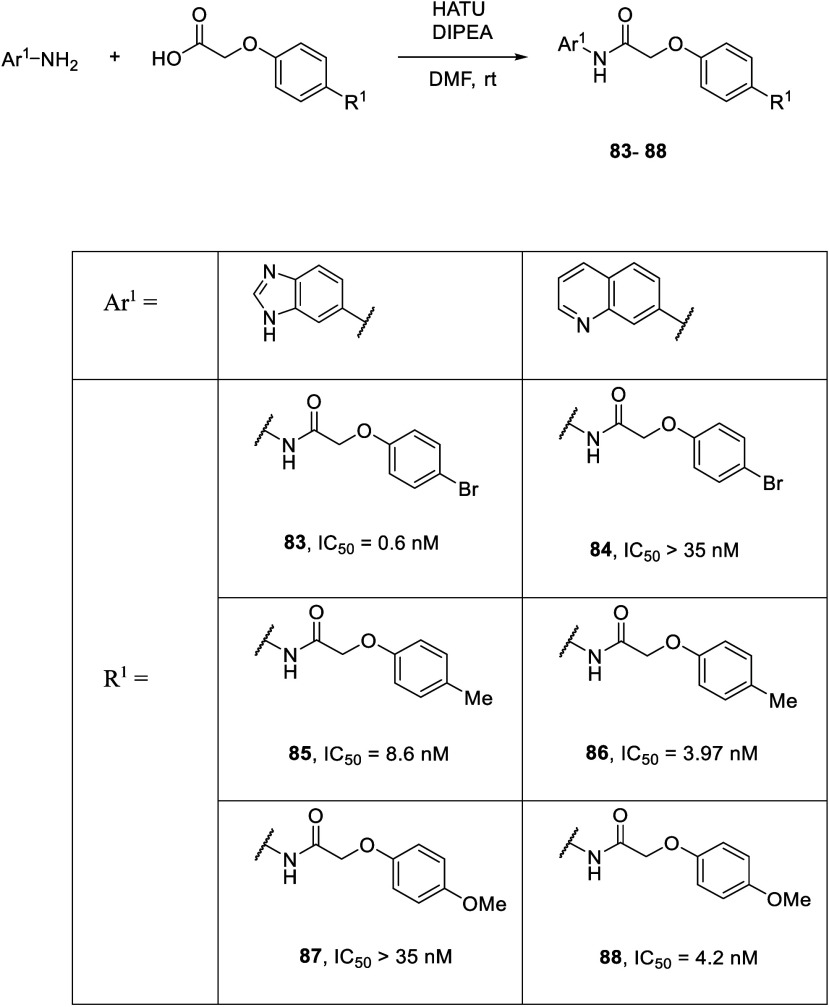
Exemplar SAR of *N*-Heteroaryl Phenoxy Acetamide Derivatives as P2Y_14_R Antagonists

Among them, compound **83** (IC_50_ = 0.6 nM) exhibited high P2Y_14_R selectivity (IC_50_ > 80 μM for P2Y_1,2,4,6,12_) when screened against other P2Y receptor subtypes and showed excellent stability in human liver microsomes and moderate bioactivity (*F* = 75%, 20 mg/kg, po) in rats (Table S2). Compound **83** showed extremely potent anti-inflammatory effects *in vitro* and *in vivo*. The anti-inflammatory evaluation of **83** in the MSU-induced gout model demonstrated its protective role in joint inflammation by decreasing inflammatory factor release and cell pyroptosis through the NOD-like receptor family pyrin domain-containing 3 (NLRP3)/gasdermin D (GSDMD) signaling pathway.^135^ In addition, the high potency, neutral character, high selectivity, oral bioactivity, and improved PK/PD profiles of compound **83** are promising for further progress.

The P2Y_14_R has been identified and validated as a potential target for inflammatory conditions. Inhibition of this receptor reduced inflammation and symptoms associated with gout, neuropathic pain, asthma, and peritonitis in animal models. Preclinical studies have shown that many of these P2Y_14_R antagonists demonstrate favorable safety profiles. Indeed, the development of selective antagonists has opened new avenues for investigating this receptor’s functions and potential clinical applications. However, there are no P2Y_14_R antagonists approved or undergoing clinical trials for any conditions yet. A range of PPTN-like as well as recent acetamide derivatives are available for further preclinical and clinical studies. It will be interesting to see if the rising academic research interest in this target will encourage industrial groups to pursue targeting of this interesting P2Y receptor.

## Nonselective P2Y Receptor Antagonists

**Figure 34:**
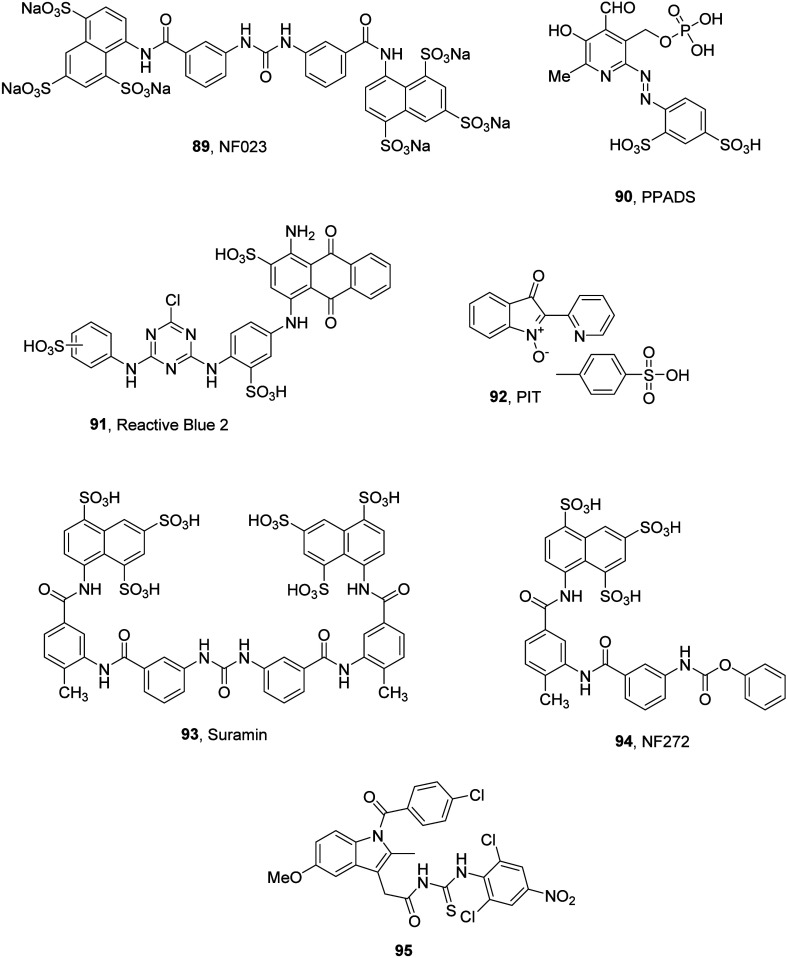
Nonselective P2YR antagonists.

There are a few nonselective low-potency non-nucleotide P2 receptor antagonists such as NF023 (**89**), PPADS (**90**), and Reactive Blue 2 (**91**) that are used in pharmacological studies.^136^ 2,2′-Pyridylisatogen tosylate (**92**, PIT), an isatogen analogue, is a subtype-selective allosteric modulator of the P2Y_1_R with an IC_50_ value of 0.14 μM. It also demonstrates low affinity for a range of GPCRs, including the adenosine (A_1_) receptor with a *K*_i_ value of 5.01 μM.^137^ Suramin (**93**), an antiparasitic drug, antagonizes a range of P2 receptors, including the P2Y_2_R, with high micromolar potency (IC_50_ = 796 μM).^138^ Screening a library of 415 suramin-derived compounds at the P2Y_2_R identified NF272 (**94**, IC_50_ = 58 μM), which retains half of the suramin structure while the urea is replaced by a phenyl-carbamate. Potency was determined using a fluorescence-based assay measuring the inhibition of UTP- or ATP-induced intracellular Ca^2+^ release in 1321N1 astrocytoma cells stably transfected with the P2Y_2_R. However, NF272 had higher affinity for the P2Y_11_ and P2Y_12_ receptors while also antagonizing the P2Y_1_R.^138^ Using a calcium mobilization assay following receptor activation with UDP in 1321N1 astrocytoma cells recombinantly expressing P2Y_1,2,4,6_ receptors, Bano et al. reported the identification of a small series of indomethacin-derived thioureas as inhibitors of the P2Y_1_, P2Y_2_, P2Y_4_, and P2Y_6_ receptors. The series most potent compound, **95**, exhibited a P2Y_1_R IC_50_ value of 0.36 μM and had modest selectivity against other subtypes of P2Y receptors (P2Y_2_R IC_50_ = 5.39 mM and 43% and 48% inhibition of the P2Y_4_R and P2Y_6_R, respectively, at 100 μM). The compounds contain acylated thioureas, which could potentially be reactive and limit their usefulness as *in vivo* tools. To alleviate concerns, a cell viability study was carried out to assess potential cytotoxicity. No significant cell toxicity was observed when the most potent analogues were assayed up to 100 μM, but no metabolism studies were reported (Figure 34).^139^

## Conclusion

The role of P2Y receptors in various physiological and pathological processes has been further illuminated in recent years. Their wide distribution in almost all mammalian tissues and organs underlines the therapeutic relevance of this class of receptors.^57^ Development of subtype-specific inhibitors of P2Y receptors is key to modulate their cellular effects for research and clinical applications.^37^ P2Y_12_R-specific antagonists, both irreversible and reversible, have been a huge success in preventing and treating cardiac-associated conditions. Among them, clopidogrel has featured in multiple lists as one of the most prescribed medications. Selatogrel, another selective P2Y_12_R inhibitor, has shown positive results in recent phase 2 clinical trials when administered subcutaneously.^64^ However, despite the abundance of pharmacological data showing potential clinical applications, there are no marketed drugs or candidates in the late stages of clinical trials targeting other P2Y subtypes. The availability of antagonist-bound X-ray crystal structures for both G_q_-coupled and G_i_-coupled subtypes is guiding the development of novel selective inhibitors.^3,5^

The P2Y_1_R has shown promise as an antithrombotic target with decreased bleeding risk. Drug-like inhibitors based on biaryl urea scaffolds have been investigated vigorously as antiplatelet medications, but the challenges associated with physicochemical properties hindered the oral administration of this class.^16^ The X-ray crystal structure of the BPTU-bound receptor highlighted the unique allosteric binding mode of this chemotype. Moreover, the binding mode of nucleotide-like MRS2500 is also available to guide the structure-based drug design. The recent developments in this area include the development of dual inhibitors of the P2Y_1_ and P2Y_12_ receptors as antithrombotics with decreased side effects, as well as an emerging interest in the CNS applications of P2Y_1_R ligands as PET tracers and brain-penetrant inhibitors.^29^ With the availability of three-dimensional structures as well as conventional and allosteric antagonists in hand, P2Y_1_R is an attractive target for drug discovery programs. Further research on the biased antagonism and its therapeutic relevance would also be appealing.

AR-C118925 is the most used pharmacological tool for *in vitro* and *in vivo* studies of the P2Y_2_R. The commercial availability of this compound has facilitated numerous studies to understand the therapeutic potential of the P2Y_2_R. Unsurprisingly, these studies reiterate P2Y_2_R’s relevance and potential applications in various inflammatory conditions and cancer. Due to the poor oral bioavailability of AR-C118925, novel drug-like P2Y_2_R antagonists are in demand. Our group’s ongoing research developing novel P2Y_2_R antagonists and fluorescent ligands for applications in pharmacological assays (e.g., NanoBRET assays) will hopefully encourage medicinal chemistry efforts targeting the P2Y_2_R.^38,44,45^

The P2Y_14_R has attracted significant interest in recent years. Structural optimization of highly potent and selective but orally unavailable zwitterionic antagonist PPTN has provided multiple lead compounds with high potency and *in vivo* efficacy. Novel compounds developed thus far have shown promising efficacy in animal models of inflammatory conditions such as gout by inhibiting NLRP3 inflammasome activation. Complementary to functional assays, P2Y_14_R-selective fluorescent probe-based assays have also been developed for quantifying binding affinities.^57,113^

SBVS using MM/GBSA free energy calculations has provided novel *in vivo* efficacious drug-like antagonists of the P2Y_14_ and P2Y_6_ receptors, underlining the importance of the X-ray crystal structures resolved for the P2Y_1_ and P2Y_12_ receptors. Newly discovered acidic and neutral P2Y_6_R-selective antagonists allowed proof-of-concept studies demonstrating the anti-inflammatory potential of this subtype.^52,131,135^ In our opinion, currently there are no oral drug-like antagonists of the P2Y_4_, P2Y_11_, and P2Y_13_ receptors. However, there are subtype selective tool compounds such as PSB16133, NF340, and MRS2603 available for the pharmacological characterization of these receptors.

For many of the novel selective drug-like antagonists summarized in this Perspective, PK properties including solubility, half-life, clearance, and volume of distribution were evaluated to confirm their drug-likeliness. Moreover, the risk of toxicity, drug interactions (CYP inhibition), and cardiac toxicity (*h*ERG inhibition) were also studied to make sure the candidates are safe. Where relevant, *in vivo* efficacy was evaluated using animal models of thrombosis and bleeding time, as well as various mouse models of inflammation. Several novel subtype selective inhibitors of P2Y receptors have proven to be safe and efficacious, thus warranting further studies.

## Perspective Comment

Despite promising preclinical results, many P2YR antagonists do not enter clinical evaluation, and the clinical pipeline for P2YR antagonists demonstrates this remains a challenge, as there are currently clinical trials progressing for P2Y_12_R antagonists alone.^140,141^ In addition, to the best of our knowledge, there are no P2Y_1,2,4,6,11,13,14_ receptor antagonists reported to be progressing into early clinical evaluation as oral drug candidates.

Despite the extensive preclinical evidence for the role of P2YR antagonists in a multitude of potential clinical applications, it is interesting to note that the only orally bioavailable clinically approved compounds appear to target P2Y allosteric binding sites. This is due to most studies targeting the highly charged orthosteric binding site through modification of the agonist nucleotide structure. While this approach has proven successful in delivering proof of concept tool compounds and iv administered drugs, functional group limitations imposed on the resulting P2YR antagonists by binding site requirements, such as high polarity and ionized functional groups, have made obtaining compounds showing oral drug-like properties challenging.^13,38,71^ This has led to P2YR inhibitors that target the orthosteric P2YR sites suffering a combination of poor bioavailability or metabolic instability, limiting their use to *iv* administration alone. Strategies such as ester prodrug approaches and isosteric replacements have been explored to overcome these limitations. While these approaches have enabled concept validation in preclinical models, their successful translation into clinical use remains a challenging.

A further potential issue with the nucleotide-based P2YR ligands is that most of the published research in the P2YR field makes little effort to confirm *in vitro*, *in vivo* circulating, or *in vivo* target tissue concentrations of nucleotide P2 receptor ligands. In a recent report of *in vitro* and *in vivo* stability, alongside limited pharmacokinetic studies, prototypical nucleotide P2Y_1_R agonists and antagonists, including the constrained ring analogue (MRS2500) which was reported to form more slowly hydrolyzed nucleotides compared to the riboside analogues, were studied. However, *in vitro* incubations in mouse and human plasma and whole blood demonstrated rapid hydrolysis to the corresponding nucleoside metabolite, which was reported to be far less active at the P2Y_1_R. Therefore, given the likely susceptibility of further phosphorylated nucleotides to hydrolysis, it is critically important to ensure that effects attributed to P2YR antagonists (and agonists) are indeed due to the interactions with the P2YR and not the released nucleoside metabolites, which may possess inherent polypharmacology. A further complication in the development of nucleotide-based P2YR ligands is that the rate of dephosphorylation was shown to be species-dependent. This has issues for compounds entering preclinical development, as it is likely that results obtained from *in vitro* and *in vivo* studies might not scale to pharmacokinetic and dose predictions for human safety and efficacy studies. Finally, the formation of interspecies metabolites with their own pharmacology could be problematic in analyzing efficacy and safety data. The study also concludes with the importance of using non-nucleotide-based P2YR antagonists and/or mouse knock-down models to fully understand *in vivo* pharmacology for the above reasons.^142^

Recent and extensive investigation of purinergic signaling has led to the suggestion that some of the early P2YR antagonists, such as suramin, Reactive Blue 2, and PPADS, might not attribute their pharmacological activity solely to antagonism of specific P2YR-mediated mechanisms but instead to off-target effects. Indeed, these compounds are now thought to bind and interact with other enzymes and proteins leading to non-P2YR-specific effects.^143^

The search for small-molecule P2YR-selective inhibitors, with confirmed binding, is therefore of importance, as functional assays, such as widely employed agonist activated calcium mobilization assays, can become misleading due to off-target activity and/or fluorescence interference, leading to false positives and negatives.^144^ Therefore, confirmation of P2Y receptor affinity is of importance to ensure off target activities are not driving the observed functional P2YR inhibition and the application of chemical biology tools in combination with highly sensitive screening methods capable of determining low-affinity P2YR inhibitors should be explored alongside structural studies if available. Recent advances in screening technologies and the application of structural information should accelerate the identification of new allosteric ligands with confirmed selective P2YR binding, such as those reported for the P2Y_2_R and P2Y_6_R, and we eagerly await further progress reports in these areas.

It is noteworthy that the preclinical therapeutic potential for P2YR antagonists continues to expand alongside building evidence for P2YR inhibitors having CNS disease applications as specific P2Y subtypes, like P2Y_1_, P2Y_2_, P2Y_4_, P2Y_6_, and P2Y_12_ expressed in the CNS.^1^ The role of targeting the P2Y_12_R in the central nervous system, where it is expressed exclusively on microglia, has led to extensive research as a potential therapy for degenerative diseases such as multiple sclerosis and Alzheimer’s disease, driven in part by the availability of selective oral drug-like inhibitors for this P2Y subtype.^68^

In addition, the potential for P2Y_1_ receptor antagonists in Alzheimer’s disease has growing evidence and has recently been reviewed.^145^ In a noteworthy study by Boyer, osmotic minipumps were used either to infuse or intracerebroventricularly (i.c.v.) deliver the P2Y_1_R antagonist MRS2179 and demonstrated that chronic P2Y_1_R inhibition reduces neuronal–astroglial network hyperactivity in an Alzheimer’s disease (APP/PS1) transgenic mouse model. In the same study, the selective allosteric inhibitor BPTU also displayed reduced astroglial hyperactivity through osmotic minipump delivery.^146^

However, except for the P2Y_12_R, where a toolbox of oral drug-like receptor antagonists resides, challenges prevail in balancing the physicochemical properties required for oral drug delivery and resulting BBB penetration with the physicochemical property requirements for orthosteric antagonist binding at the highly charged nucleotide agonist binding site. It is therefore hoped that recently disclosed oral drug-like allosteric binding P2YR antagonists will show target validation in preclinical models, allowing progression to preclinical development. Medicinal chemistry research should accelerate toward the identification of new preclinical oral drug-like P2YR antagonists, allowing further *in vivo* concept testing in relevant disease models.

## Data Availability

All data associated with this research is available through the supporting information or via the corresponding author.

## Supporting Information Available

Further information on antagonists of P2YR, known pharmacokinetic data in rats, and compound numbers and SMILES annotation (PDF)

## Abbreviations

ADMET: absorption, distribution, metabolism, excretion and toxicity
ADP: adenosine diphosphate
ALI: acute lung injury
ASC: the adaptor molecule apoptosis associated speck-like protein containing a CARD
ATP: adenosine 5′-triphosphate
Ap4A: *P*1,*P*4-di(adenosine-5′) tetraphosphate
BPTU: *N*-[2-[2-(1,1-dimethylethyl)phenoxy]-3-pyridinyl]-*N*′-[4-(trifluoromethoxy)phenyl]urea
cAMP: cyclic adenosine monophosphate
CHO: Chinese hamster ovary
CL: clearance
CNS: central nervous system
DSS: dextran sodium sulfate
*F*: bioavailability
FLIPR: fluorescent imaging plate reader
GPCR: G protein-coupled receptor
HEK: human embryonic kidney
*h*ERG: human ether-à-go-go-related gene
*h*PRP: human platelet-rich plasma
*t*_1/2_: terminal half-life
HTS: high-throughput screening
LPS: lipopolysaccharide
MM/GBSA: molecular mechanics/generalized born surface area
MPO: multiparameter optimization
NLRP3: NLR family pyrin domain containing 3
PA: platelet aggregation
PD: pharmacodynamic
PDB: Protein Data Bank
PET: positron emission topography
PIT: 2,2′-pyridylisatogen tosylate
PK: pharmacokinetic
PPADS: pyridoxal-phosphate-6-azophenyl-2′,4′-disulfonate
PPB: plasma protein binding
PPTN: 4-[4-(4-piperidinyl)phenyl]-7-[4-(trifluoromethyl)phenyl]-2-naphthalenecarboxylic acid
SAR: structure–activity relationship
SBVS: structure-based virtual screening
UDP: uridine diphosphate
UTP: uridine-5′-triphosphate
*V*_d_: volume of distribution
