# Supplementary material for: Drug-like Antagonists of P2Y Receptor Subtypes: An Update
Source: J Med Chem. 2025 Apr 28;68(9):9057–83. doi: 10.1021/acs.jmedchem.5c00249 (PMC12067450; doi:10.1021/acs.jmedchem.5c00249)
Supplement: Supplementary file 1 — jm5c00249_si_001.pdf [file jm5c00249_si_001.pdf]

Drug-like Antagonists of P2Y Receptor Subtypes - An Update

Mahesh Puthanveedu[a], Rebecca Knight [b,c], and Michael J. Stocks[a]*

a. Division of Biomolecular Sciences and Medicinal Chemistry, Biodiscovery Institute, School of Pharmacy, University of Nottingham, Nottingham NG7 2RD, United Kingdom.

b. Division of Physiology, Pharmacology and Neuroscience, School of Life Sciences, University of Nottingham, Nottingham NG7 2UH, United Kingdom.

c. Centre of Membrane Proteins and Receptors, University of Birmingham and Nottingham, The Midlands NG7 2UH, United Kingdom.

*Corresponding Author's email address: Michael.stocks@nottingham.ac.uk

List of Contents:

Table S1: Further Information on Antagonists of P2YR	Page S2
Table S2: Pharmacokinetic data in rats	Page S15
Table S3. Compound numbers and SMILES	Page S17

Table S1: Further Information on Antagonists of P2YR

Compound	*In Vitro* data	Assay information	Binding site/mode	Status	Comments	Clinical relevance	*In Vivo*/PD data	Year & Ref.
**Antagonists of $P2Y_1R$**								
MRS2500 (**1**)	K_i = 0.79 nM	Radioligand binding assay (*h*$P2Y_1R$ expressed in Sf9 cells)	Within the 7 TM bundle<br><br>PDB ID: 4XNW	Preclinical	Nucleotide analogue	Cardiovascular<br>Neurological<br>Endocrine<br>Cancer<br>Inflammation	Strong antithrombotic activity in mice<br><br>Prevents carotid artery thrombosis in cynomolgus monkeys	[13]<br><br>2003
	IC_{50} = 0.95 nM	Inhibition of ADP-induced aggregation of human platelets						
BPTU (**2**)	K_i = 6 nM	Radioligand binding assay (*h*$P2Y_1R$ transfected into HEK-293 cells)	Allosteric<br><br>PDB ID: 4XNV	Preclinical	Non-nucleotide ligand	Cardiovascular<br>Neurological/<br>PET tracers<br>Gastrointestinal<br>Inflammation	Reduces thrombus weight in a rat model of iron chloride-induced arterial thrombosis	[14]<br><br>2013
	IC_{50} = 2.1 μM	Inhibition of platelet aggregation in human platelet-rich plasma						

Compound	*In Vitro* data	Assay information	Binding site/mode	Status	Comments	Clinical relevance	*In Vivo*/PD data	Year & Ref.
BMS-884775 (**3**)	IC_{50} = 0.12 nM	FLIPR (Fluorescent Imaging Plate Reader) assay	Allosteric	Preclinical	Non-nucleotide ligand	Cardiovascular	Demonstrated similar antithrombotic efficacy with less bleeding compared with prasugrel in rabbit efficacy and bleeding models	[15] 2014
	IC_{50} = 0.18 μM	Inhibition of platelet aggregation in human platelet-rich plasma						
**4**	IC_{50} = 0.21 μM	Intracellular calcium assay (1321N1 cells stably expressing $P2Y_1R$)	Allosteric	Preclinical	Non-nucleotide ligand (BPTU derivative)	Cardiovascular	69% inhibition of ADP-induced platelet aggregation in rats (*ex vivo* data)	[20] 2018
**Dual $P2Y_1$ and $P2Y_{12}$ Antagonists**								
GLS-409 **(5)**	$P2Y_1R$ IC_{50} = 2.15 μM	$P2Y_1$-mediated increase in platelet cytosolic Ca^{2+} (measured using flow cytometry)		Preclinical	diadenosine tetraphosphate (Ap_4A) analogue<br><br>$P2Y_1R$ and $P2Y_{12}R$ selectivity	Cardiovascular	Rapid improvement in coronary patency in a canine model of *in vivo* platelet-mediated thrombosis without an associated	[24] 2016
	$P2Y_{12}R$ IC_{50} = 0.22 μM	$P2Y_{12}$-mediated changes in VASP phosphorylation (analysed using flow cytometry)						

Compound	*In Vitro* data	Assay information	Binding site/mode	Status	Comments	Clinical relevance	*In Vivo*/PD data	Year & Ref.
							increase in the bleeding time at low doses	
**7a**	$P2Y_1R$ IC_{50} = 2.59 μM	$P2Y_1$-mediated increase in platelet cytosolic Ca^{2+} (measured using flow cytometry)	Nucleotide binding site (predicted)	Preclinical	Small molecule	Cardiovascular	Demonstrated comparable antithrombotic efficacy to that of ticagrelor in a rat ferric chloride model with significantly lower bleeding weight and time	[26] 2020
	$P2Y_{12}R$ IC_{50} = 148 μM	$P2Y_{12}$-mediated changes in VASP phosphorylation (analysed using flow cytometry)						
**Antagonists of $P2Y_2R$**								
**15**	pA_2 = 8.5	Intracellular fluorescence-based calcium mobilisation assay (*h*$P2Y_2R$-Jurkat cells)	Antagonist	Preclinical	Small molecule	Cardiovascular Cancer Inflammation		[37] 2017
**16**	pA_2 = 4.7							
**17**	pA_2 = 7.0	Calcium mobilisation assay (*h*$P2Y_2R$-expressing Jurkat cells)	Antagonist	Preclinical	Small molecule	Cardiovascular Cancer Inflammation		[37] 2017
AR-C 118925 (**18**)	pA_2 = 7.0	Calcium mobilisation	Antagonist	Preclinical	Small Molecule	Cardiovascular		[37]

Compound	*In Vitro* data	Assay information	Binding site/mode	Status	Comments	Clinical relevance	*In Vivo*/PD data	Year & Ref.
		assay (*h*P2Y$_2$R-expressing Jurkat cells)				Cancer Inflammation		2017
	K_b = 30.9 nM	Calcium mobilisation assay (*h*P2Y$_2$R-expressing 1321N1 cells)	Antagonist	Preclinical	Small Molecule	Cardiovascular Cancer Inflammation		[44] 2018
	K_i = 41.7 nM	NanoBRET fluorescent ligand binding assay (*h*NLuc-P2Y$_2$R-expressing 1321N1 cells)						
**19**	IC_{50} = 9.26 µM	Calcium mobilisation assay (*h*P2Y$_2$R-expressing 1321N1 cells)	Antagonist	Preclinical	Small Molecule	Cardiovascular Cancer Inflammation		[40] 2022
**20**	IC_{50} = 9.87 µM							
**21**	IC_{50} = 10.9 µM							
**22**	IC_{50} = 3.43 µM	Calcium mobilisation assay (*h*P2Y$_2$R-expressing 1321N1 cells)	Antagonist	Preclinical	Small Molecule	Cardiovascular Cancer Inflammation		[42] 2020
**23**	IC_{50} = 3.17 µM							
**24**	IC_{50} = 3.01 µM							
**25**	K_b =		Antagonist	Preclinical	Small Molecule			

Compound	*In Vitro* data	Assay information	Binding site/mode	Status	Comments	Clinical relevance	*In Vivo*/PD data	Year & Ref.
	0.33 μM	Calcium mobilisation assay (*h*P2Y$_2$R-expressing 1321N1 cells)				Cardiovascular Cancer Inflammation		[44] 2018
**26**	K_b = 1.02 μM							
**27**	K_b = 0.16 μM							
**30**	K_i = 1 mM	NanoBRET fluorescent ligand binding assay (*h*NLuc-P2Y$_2$R-expressing 1321N1 cell membranes)	Antagonist	Preclinical	Small Molecule	Cardiovascular Cancer Inflammation		[45] 2024
**31**	K_i = 6.76 μM							
**32**	K_d = 0.95 μM				Fluorescent Ligand			
**Antagonists of P2Y$_4$R**								
**33**	P2Y$_4$R IC_{50} = 233 nM	Calcium Mobilisation in 1321N1 astrocytoma cells	Allosteric	Preclinical	Small Molecule	Wide distribution in the body, including heart, GIT, CNS, skin, and ear		[47] 2017
**Antagonists of P2Y$_6$R**								
TIM-38 (**34**)	IC_{50} = 2.91 μM	Calcium Mobilisation (*h*P2Y$_6$R-expressing 1321N1 cells)	Surmountable antagonist	Preclinical	Small Molecule	Inflammation, CNS, Metabolic diseases		[49] 2017

Compound	*In Vitro* data	Assay information	Binding site/mode	Status	Comments	Clinical relevance	*In Vivo*/PD data	Year & Ref.
**35**	IC_{50} = 0.78 µM	Calcium Mobilisation (*h*P2Y$_6$R-expressing 1321N1 cells)	Surmountable antagonist	Preclinical	Small Molecule	Inflammation, CNS, Metabolic diseases		[50] 2021
**36**	IC_{50} = 0.60 µM							
MRS4853 (**37**)	IC_{50} = 0.46 µM							
**38**	IC_{50} = 0.13 µM	Calcium Mobilisation (*h*P2Y$_6$R-expressing 1321N1 cells)	Unknown	Preclinical	Small Molecule	Inflammation, CNS, Metabolic diseases		[51] 2022
**39**	IC_{50} = 5.91 nM	Calcium Mobilisation	Unknown (predicted pose available)	Preclinical	Small Molecule	Inflammatory diseases such as IBD, IPF, ALI.	Ameliorate DSS-induced ulcerative colitis in mice through inhibiting the activation of NLRP3 inflammasome in colon tissues. Reduced LPS-induced pulmonary edema and infiltration of	[52] 2023
	IC_{50} = 3.47 µM	P2Y$_6$ grating-coupled interferometry binding assay						

**Compound**	***In Vitro* data**	**Assay information**	**Binding site/mode**	**Status**	**Comments**	**Clinical relevance**	***In Vivo*/PD data**	**Year & Ref.**
							inflammatory cells in mice	
**40**	IC_{50} = 19.6 nM	Calcium Mobilisation (CHO-h$P2Y_6R$)	Unknown<br><br>(predicted pose available)	Preclinical	Small Molecule	Intestinal inflammation, tracheal inflammation and diabetes	Attenuated LPS-induced acute lung injury in mice	[53]<br><br>2024
**Antagonists of $P2Y_{11}R$**								
NF340 (**41**)	K_i = 44.3 nM	Fluorescence calcium assay (In $P2Y_{11}R$ expressed in 1321N1 astrocytoma cells & HEK293 cells endogenously expressing $P2Y_1R$ & $P2Y_2R$		Preclinical	Suramin derivative<br><br>(selective to $P2Y_{11}$ among P2YRs)	Inflammation and neuropathic pain	NF340 ameliorates inflammation in human fibroblast-like synoviocytes (*ex vivo*)	[58]<br><br>2005
**Antagonists of $P2Y_{12}R$**								
ticlopidine (**42**)			Prodrug, Covalent binder	Marketed drug	Oral tablets, 250 mg BID	Indicated for patients who cannot take aspirin or in whom aspirin has not worked to prevent	Onset within 4 days<br>$t_{1/2}$ = 12.6 h (single dose)<br>$t_{1/2}$ = 4-5 days (BID)	[69]<br><br>1991 (FDA approval)

Compound	*In Vitro* data	Assay information	Binding site/mode	Status	Comments	Clinical relevance	*In Vivo*/PD data	Year & Ref.
						a thrombotic stroke.		
clopidogrel (**43**)			Prodrug, Covalent binder	Marketed drug	Oral tablets 300 mg (LD)<br>75 mg (QD)	Better safety profile (reduced bleeding risk) compared to ticagrelor	Onset of action in 2 h<br>$t_{1/2}$ = 8 h	[69]<br><br>1997 (FDA approval)
prasugrel (**44**)			Prodrug, Covalent binder	Marketed drug	Oral tablets, 60-80 mg (LD)<br>10 mg (QD)	Indicated to prevent atherothrombotic events in patients with acute coronary syndrome (ACS)	Onset of action in < 0.5 h<br>$t_{1/2}$ = 3.7 h	[70]<br><br>2009(FDA approval)
ticagrelor (**45**)			Allosteric<br><br>Reversible binder	Marketed drug	Oral tablets,<br><br>180 mg (LD)<br>60 mg (BID)<br>90 mg (BID)	Used in patients with a history of myocardial infarction (MI) or with acute coronary syndrome	0.5 h (40% inhibition)<br>1 h (80% inhibition)<br>$t_{1/2}$ = 7 h (ticagrelor)<br>$t_{1/2}$ = 9 h (active metabolite)	[71]<br><br>2011 (FDA approval)
cangrelor (**46**)			Reversible binder	Marketed drug	30 mcg/kg IV bolus for 1 min before PCI, then 4 mcg/kg/min IV infusion for at least 2 hr	Intravenous, direct-acting, reversible drug for patients undergoing percutaneous	Onset of action in<br>< 2 min<br>$t_{1/2}$ = 3-6 min	[72]<br><br>2015 (FDA approval)

Compound	*In Vitro* data	Assay information	Binding site/mode	Status	Comments	Clinical relevance	*In Vivo*/PD data	Year & Ref.
						coronary intervention (PCI)		
elinogrel (**47**)	IC_{50} < 0.05 µM	Radioligand binding assay	Reversible binder	Phase 2: not progressed (reason not disclosed)	Non-nucleotide analogue	Cardiovascular	Dose dependent antiplatelet effect in mice and low impact on tail bleeding time	[74] 2008
	IC_{50} <10 µM	Inhibition of ADP-induced aggregation of human platelets						
SAR216471 (**48**)	IC_{50} = 17 nM	Radioligand binding assay ($P2Y_{12}R$-expressing CHO cells)	Reversible binder	Preclinical	Non-nucleotide antagonist	Cardiovascular	Rat shunt thrombosis model demonstrated a dose-dependent antithrombotic activity after oral administration.	[75] 2014
	IC_{50} = 0.10 µM	Inhibition of platelet aggregation in human platelet-rich plasma						
AZD1283 (**49**)	IC_{50} = 0.01 µM	Radioligand binding assay ($P2Y_{12}R$-expressing CHO cells)	Pocket 1 composed of helices III–VII<br>PDB ID: 4NTJ	Phase I<br>(discontinued due to low	Non-nucleotide antagonist	Cardiovascular	>10 times separation of bleeding and antithrombotic effect in	[78] 2013

Compound	*In Vitro* data	Assay information	Binding site/mode	Status	Comments	Clinical relevance	*In Vivo*/PD data	Year & Ref.
	IC_{50} = 0.025 μM	Functional GTPγS assay ($P2Y_{12}R$-expressing CHO cells)		uptake and high metabolism)			modified Folts dog model	
**53d**	IC_{50} = 2.94 μM	Inhibition of platelet aggregation in human platelet-rich plasma	AZD1283 like (proposed)	Preclinical	Non-nucleotide antagonist (AZD1283 derivative)	Cardiovascular	Inhibition of platelet aggregation *in vivo* ($FeCl_3$-induced carotid artery thrombosis model)<br><br>decreased bleeding time and weight compared with clopidogrel.	[80]<br><br>2019
BX-667 (**56**)	IC_{50} = 97 nM	Inhibition of ADP-induced platelet aggregation in human, dog and rat blood	Reversible inhibitor	Preclinical	Non-nucleotide antagonist (piperazinyl carbamate class)	Cardiovascular	High oral bioavailability in both dog and rat	[81]<br><br>2007
Selatogrel (**58**)	K_d = 1.5 nM	Radioligand binding assay	AZD1283 like	Phase 3 (SOS-AMI)	Non-nucleotide antagonist	Prevent thrombus development in	Quick onset/offset and	[86]

Compound	*In Vitro* data	Assay information	Binding site/mode	Status	Comments	Clinical relevance	*In Vivo*/PD data	Year & Ref.
		(CHO cells expressing recombinant $P2Y_{12}$ receptors)	PDB ID: 7PP1	(NCT04957719)	(piperazinyl carbamate class)<br><br>Subcutane-ous (sc)	the critical first few hours of a cardiac emergency	potentially self-administrable antiplatelet agent	2015
	IC_{50} = 14 nM	Inhibition of ADP-induced platelet aggregation						
**Antagonists of the $P2Y_{13}$ R**								
MRS 2211<br><br>MRS 2603	pIC_{50} = 5.97<br><br>pIC_{50} = 6.18	Inhibition of ADP induced inositol trisphosphate (IP_3) formation<br><br>(Recombinant *h*$P2Y_{13}$R-AG32 in 1321N1 human astrocytoma cells)		Preclinical	PPADS analogues	Inflammation Neuropathic- pain Lipid- metabolism		[112]<br><br>2005
**Antagonists of $P2Y_{14}$R**								

Compound	*In Vitro* data	Assay information	Binding site/mode	Status	Comments	Clinical relevance	*In Vivo*/PD data	Year & Ref.
PPTN (**67**) & prodrug **68**	K_i = 35 nM (**67**)	Chimpanzee $P2Y_{14}$ (in presence of 2% HSA)		Preclinical	non nucleotide analogues	Inflammatory diseases		[118] 2011
**73**	K_i = 3.44 nM<br><br>IC_{50} = 5.92 nM	*h*$P2Y_{14}$R affinity in a fluorescent whole-cell competitive binding assay		Preclinical	PPTN derivative	Inflammatory diseases<br><br>Neuropathic pain	Reduced airway eosinophilia in a protease-mediated asthma model (po)<br><br>reversed chronic neuropathic pain (po, mouse CCI model).	[126] 2023
**75**	IC_{50} = 2.18 nM	Inhibition based on the production of cAMP in a HEK293 cell line stably expressing $P2Y_{14}$R.		Preclinical	PPTN analogue	Inflammatory diseases	Efficiently reversed the paw swelling and infiltration of inflammatory cells in foot tissues (MSU-induced mice paw swelling model)	[128] 2021

Compound	*In Vitro* data	Assay information	Binding site/mode	Status	Comments	Clinical relevance	*In Vivo*/PD data	Year & Ref.
**77**	IC_{50} = 0.40 nM	Functional assay to (antagonism of agonist-induced inhibition of cAMP production)	Predicted pose	Preclinical	PPTN bio-isosteric analogue	IBD and other inflammatory disorders	Remarkable anti-IBD effect even at low doses (experimental colitis mouse model)	[129] 2024
**81**	IC_{50} = 2 nM	cAMP functional assay (HEK293 cell lines stably expressing $P2Y_{14}R$)	Predicted pose	Preclinical	Small molecule (neutral)	Gout and other inflammatory disorders	Exhibits *in vivo* anti-gout activity in MSU-induced acute gouty arthritis mice model through cAMP/ NLRP3/GSDMD signalling pathways.	[133] 2022
**83**	IC_{50} = 0.6 nM	Antagonism of the agonist-induced inhibition of cAMP production in the presence of forskolin (30 mM) in a HEK293 cell line stably expressing $P2Y_{14}R$	Predicted pose	Preclinical	Small molecule (neutral)	acute gouty arthritis and other inflammatory disorders	Satisfactory inhibitory activity on the inflammatory response of MSU-induced acute gouty arthritis mouse model	[135] 2024

Table S2: Pharmacokinetic data in rats

**Compound**	**Dosage**	C_{max}	t_{max}	**AUC total**	*CL*	V_d	t_{half}	*F*
BPTU **(2)**	iv: 1 mg/kg, po: 30 mg/kg	5830 nM	2.0 h	15900 nM·h	13 mL/min/kg	0.8 L/kg	1.4 h	18%
BMS-884775 (**3**)	iv				4 mL/min/kg	2.1 L/kg		30%
**7a**	po: 10 mg/kg	67.99 ng/ml	0.5 h	1135.08 ng·h/mL	12.40 $L \cdot h^{-1} \cdot kg^{-1}$	190.6 L/kg	13.8 h	32%
AR-C118925 (**18**)					75 mL/min/kg	4.34 L/kg	2.12 h	0 %
SAR216474 (**48**)	po: 10 mg/kg	168 ng/ml	1 h	1300 ng.h/ml			3.7 h	18%
AZD1283 (**49**)	Po: 5 mg/kg	25.9 ng/ml	0.25 h	34.0 ng.h/ml				
**53d**	Po: 5 mg/kg	1661 ng/ml	0.25 h	4120 ng.h/ml			3 h	51%

Compound	Dosage	C_{max}	t_{max}	AUC total	*CL*	V_d	t_{half}	*F*
BX-667 (**56**)	Po: 2 mg/kg	1.9 µM	1 h	6.9 µg·h/mL				57%
**39**	iv: 2mg/kg	1054 ng/mL	34.5 min	260.55 ng·h/mL	129.65 mL/min/kg		1.8 h	
	po: 20 mg/kg	53.68 ng/mL	120 min	415.17 ng·h/mL	415.02 mL/min/kg		6.5 h	16%
**40**	iv: 10 mg/kg	1406 ng/mL	28 min	1956 µg/L.h	6.1 mL/min/kg			
	po 20 mg/kg	2943 ng/mL	46 min	2982 µg/L.h	28.3 mL/min/kg			43%
PPTN (**67**)	po 50 mg/kg	0.68 µM		23 µM h				5%
**68**	po 50 mg/kg	2.28 µM		54 µM h	1.6 mL/min/kg			67% (virtual)
**73**	po 10 mg/kg	2020 ng/mL	4 h	14400 ng·h/mL	685 mL/h/kg	4090 mL/kg	4.1 h	114%
**75**	po 10 mg/kg	1801 ng/mL		436599.5 min. ng/mL	6.03 mL/min/kg	212.67 mL/kg	2.5 h	48%
**77**	po 5 mg/kg	518 ng/mL	15 min	90744 min. ng/mL	232 mL/min/kg		3 h	65%

Compound	Dosage	C_{max}	t_{max}	AUC total	*CL*	V_d	t_{half}	*F*
**83**	po 20 mg/kg	2895 ng/mL	32 min	2845 µg/L.h	15 mL/min/kg		13 h	75%

Table S3. Compound numbers and SMILES

Compound	SMILES
Antagonists of $P2Y_1R$	
**1**	OP(O[C@@H]1[C@]2(COP(O)(O)=O)C[C@]2([H])[C@@H](N3C=NC4=C(NC)N=C(I)N=C34)C1)(O)=O
**2**	O=C(NC1=CC=C(OC(F)(F)F)C=C1)NC2=CC=CN=C2OC3=CC=CC=C3C(C)(C)C
**3**	OC1=CC(F)=C(C2=CC=C(F)C=C2)C3=C1N(C4=C(NC(NC5=NC6=C(N=C(Cl)C=C6)S5)=O)C=CC=C4)CC37CCN(CC(C)(C)C)CC7
**4**	O=C(NC1=CC=C(OC(F)(F)F)C=C1)NC2=CC=C(C)N=C2OC3=CC=CC=C3C(C)(C)C
**5**	NC1=C2C(N([C@H](O3)[C@H](O)[C@H](O)[C@H]3COP([S-])(OP([O-])(C(Cl)P(OP(OC[C@@H]4[C@@H](O)[C@@H](O)[C@H](N5C6=NC(SC)=NC(N)=C6N=C5)O4)([S-])=O)([O-])=O)=O)=O)C=N2)=NC(SC)=N1
**7**	O=C(O)C1=C(C)N=C(C2=CC=CC(C#N)=C2)N1OCC3=CC=C(C#N)C=C3
**7a**	O=C(OCC)C1=C(C)N=C(C2=CC=CC(C#N)=C2)N1OCC3=CC=C(C#N)C=C3

Compound	SMILES
**8**	O=C(NC1=CC=C(CCF)C=C1)NC2=CC=CN=C2OC3=C(C(C)(C)C)C=CC=C3
**14**	O=C(NC1=CC=C(CC[18F])C=C1)NC2=CC=CN=C2OC3=C(C(C)(C)C)C=CC=C3
Antagonists of $P2Y_2R$	
**15**	S=C(N1)C(C2C(C=CC=C3)=C3C=CC4=C2C=CC=C4)=CN([C@H]5OC@HC@@H[C@H]5O)C1=O
**16**	S=C(N1)C(C2C(C=CC=C3)=C3C=CC4=C2C=CC=C4)=CN([C@H]5OC@HC@@H[C@H]5O)C1=O
**17**	CC1=CC2=C(C=C1)C(C(C(N3)=S)=CN(CC4=CC=C(C(O)=O)O4)C3=O)C5=C(C=C(C)C=C5)C=C2
**18**	CC1=CC2=C(C=C1)C(C(C(N3)=S)=CN(CC4=CC=C(C(NC5=NN=NN5)=O)O4)C3=O)C6=C(C=C(C)C=C6)C=C2
**19**	CC(NC(C1=CC=CC=C1SCC(NC2=CC=CC(C#N)=C2)=O)=O)C3=NC4=CC=CC=C4S3
**20**	FC1=CC=C(/N=C2N(CC3=CC=CC=C3)C(CC(C(NC4=CC=C(C(O)=O)C=C4)=O)S/2)=O)C=C1
**21**	OC1=CC=CC(C2CC(C3=C(O)C=C(C)OC3=O)=NC4=CC(C(F)(F)F)=CC=C4S2)=C1
**22**	CC(NC(C1=CC=CC=C1SCC(NC2=CC=CC(C#N)=C2)=O)=O)C3=NC4=CC=CC=C4S3
**23**	FC1=CC=C(/N=C2N(CC3=CC=CC=C3)C(CC(C(NC4=CC=C(C(O)=O)C=C4)=O)S/2)=O)C=C1
**24**	OC1=CC=CC(C2CC(C3=C(O)C=C(C)OC3=O)=NC4=CC(C(F)(F)F)=CC=C4S2)=C1
**25**	ClC1=CC2=C(C=C1)C(C(C(N3)=S)=CN(CC4=CC=C(C(NC5=NN=NN5)=O)O4)C3=O)C6=C(SC(C)=N6)C=C2
**26**	ClC1=CC2=C(C=C1)C(C(C(N3)=S)=CN(C)C3=O)C4=C(SC(C)=N4)C=C2
**27**	ClC1=CC2=C(C=C1)C(C(C(N3)=S)=CN(C)C3=O)C4=C(SC(NCCOCC)=N4)C=C2
**28**	ClC1=CC2=C(C=C1)C(C(C(N3)=S)=CN(CC4=CC=C(C(NC5=NN=NN5)=O)O4)C3=O)C6=C(SC(NCCOC)=N6)C=C2
**29**	S=C(N1)C(C2C(N=C(NCCC(NCCNC(CCCCCNC(CCC3=[N+]4C(C=C3)=CC5=C(C)C=C(C)N5[B-<br>]4(F)F)=O)=O)=O)S6)=C6C=CC7=C2C=CC(Cl=C7)=CN(CC8=NC(C(NC9=NN=NN9)=O)=CS8)C1=O
**30**	ClC1=CC=C(C=C1)C(C2=CC=C(Cl)C=C2)C3=CN=C(NCCOCC)S3
**31**	ClC1=CC=C(C=C1)C(C2=CC=C(Cl)C=C2)C3=CN=C(NCCCCCCC(O)=O)S3
**32**	ClC1=CC=C(C=C1)C(C2=CC=C(Cl)C=C2)C3=CN=C(NCCCCC@HC(O)=O)S3
Antagonists of $P2Y_4R$	
**33**	O=C1C2=C(C(NC3=CC=C(SC4=CC(C)=C(C)C=C4)C=C3)=CC(S(=O)(O[Na])=O)=C2N)C(C5=CC=CC=C51)=O

Compound	SMILES
Antagonists of P2Y$_6$R	
**34**	FC(C(O1)C([N+]([O-])=O)=CC2=C1C=CC=C2)(F)F
**35**	C[Si](C)(C)C#CC1=CC(C=C([N+]([O-])=O)C(C(F)(F)F)O2)=C2C=C1
**36**	CC[Si](CC)(CC)C#CC1=CC(C=C([N+]([O-])=O)C(C(F)(F)F)O2)=C2C=C1
**37**	CC[Si](CC)(CC)C#CC1=CC=CC2=C1OC(C(F)(F)F)C([N+]([O-])=O)=C2
**38**	CC1=CC2=NC(C)=C(C3N(CCC(O)=O)N=C(C4=CC=CO4)C3)C=C2C=C1
**39**	CC(C)(C)N(C(C1=CC=CO1)=C2)N=C2C3=NC4=C(C=CC(Cl)=C4)N3
**40**	O=C(CCC(O)=O)N(N=C(C1=CC=CN1)C2)C2C3=CN=C(C=CC=C4)C4=C3
Antagonists of P2Y$_{11}$R	
**41**	O=C(C1=CC=C(C)C(NC(NC2=C(C)C=CC(C(NC3=CC(S(=O)([O-])=O)=CC4=CC=C(S(=O)([O])=O)C=C34)=O)=C2)=O)=C1)NC5=C6C=C(S(=O)([O-<br>])=O)C=CC6=CC(S([O-])(=O)=O)=C5.[4Na+]
Antagonists of P2Y$_{12}$R	
**42**	ClC1=CC=CC=C1CN2CCC3=C(C=CS3)C2
**43**	O=C([C@@H](N1CCC2=C(C1)C=CS2)C3=C(Cl)C=CC=C3)OC
**44**	CC(OC1=CC2=C(S1)CCN(C(C3=CC=CC=C3F)C(C4CC4)=O)C2)=O
**45**	CCCSC1=NC2=C(C(N[C@@H]3C[C@H]3C4=CC(F)=C(C=C4)F)=N1)N=NN2[C@@H]5C[C@@H]([C@H]([C@H]5O)O)OCCO
**46**	CSCCNC1=C2N=CN(C2=NC(SCCC(F)(F)F)=N1)[C@@H]3O[C@@H]([C@H]([C@H]3O)O)COP(O)(OP(O)(C(Cl)(P(O)(O)=O)Cl)=O)=O

Compound	SMILES
**47**	CNC1=CC(NC(N2C3=CC=C(C=C3)NC(NS(C4=CC=C(S4)Cl)(=O)=O)=O)=O)=C(C=C1F)C2=O
**48**	CCCC(C1=C(C)N(C2=CC=C(N=N2)NC(C3=CN(C4=C3C=C(Cl)C=C4)CC(N5CCN(CC5)C)=O)=O)N=C1)=O
**49**	O=C(OCC)C1=C(C)N=C(N2CCC(C(NS(=O)(CC3=CC=CC=C3)=O)=O)CC2)C(C#N)=C1
**50**	O=C(CCC)C1=C(SC)N=C(N2CCC(C(NS(=O)(C3(CC3)C4=CC=CC=C4)=O)=O)CC2)C(C#N)=C1
**51**	O=S(CC1=CC=CC=C1)(NC(C2CCN(CC2)C3=C(C#N)C=C(C(OC4)=O)C4=N3)=O)=O
**53d**	CC1=CC=C(C=C1)CS(=O)(NC(C2(CCN(CC2)C3=C(C#N)C=C(C(OC4)=O)C4=N3)C)=O)=O
**55**	CCOC(N1CCN(CC1)C([C@@H](NC(C2=NC3=C(C=CC=C3)C(OC)=C2)=O)CCC(O)=O)=O)=O
**56**	CCOC(N1CCN(CC1)C([C@@H](NC(C2=NC3=C(C=CC=C3)C(OC(C)(C(OCC)=O)C)=C2)=O)CCC(O)=O)=O)=O
**57**	CCOC(N1CCN(CC1)C([C@@H](NC(C2=NC3=C(C=CC=C3)C(OC(C)(C(O)=O)C)=C2)=O)CCC(O)=O)=O)=O
**58**	O=C(C1=NC(C2=CC=CC=C2)=NC(N3CC[C@@H](C3)OC)=C1)N[C@H](C(N4CCN(CC4)C(OCCCC)=O)=O)CP(O)(O)=O
**59**	O=C(OC(C)C)OCOP(C[C@@H](C(N1CCN(CC1)C(OCCCC)=O)=O)NC(C2=NC(C3=CC=CC=C3)=NC(N4CC[C@@H](C4)OC)=C2)=O)(OCOC(OC(C)C)=O)=O
**60**	O=C(OCC)C1=C(C)N=C(N2CC(N[11C](NS(C3=CC=C(Cl)S3)(=O)=O)=O)C2)C(C#N)=C1
**61**	O=C(OCC)C1=C(C)N=C(N2CCC(C(NS(=O)(CC3=CC=CC=C3)=O)=O)CC2)C([11C]#N)=C1
**62**	CCC1=CC2=C(N3CCN([11C](C4=CC=C(C5=CC=CC=C5)C=C4)=O)CC3)N=C(N6CC(NCC6)=O)N=C2S1
**63**	CCCC1=CC2=C(N3CCN4C(C3)=NN=C4C(F)(F)F)N=C(OCC[18F])N=C2S1
Antagonists of P2Y_{13}R	

Compound	SMILES
**64**	O=P(OCC1=C(C=O)C(O)=C(C)N=C1/N=N/C2=CC([N+]([O-])=O)=CC=C2Cl)(O)O
**65**	O=P(OCC1=C(C=O)C(O)=C(C)N=C1/N=N/C2=CC([N+]([O-])=O)=C(Cl)C=C2)(O)O
Antagonists of P2Y$_{14}$R	
**67**	O=C(C1=CC2=C(C=CC(C3=CC=C(C=C3)C(F)(F)F)=C2)C(C4=CC=C(C5CCNCC5)C=C4)=C1)O
**68**	O=C(N(C)C)COC(C1=CC2=C(C=CC(C3=CC=C(C=C3)C(F)(F)F)=C2)C(C4=CC=C(C5CCNCC5)C=C4)=C1)=O
**69**	O=C(C1=CC2=C(C=CC(C3=CC=C(C=C3)C(F)(F)F)=C2)C(C4=CC=C(C5CCN(CCCCC6=CN(CCCCCCNC(C7=CC(C([O-])=O)=C(C=C7)C8=C(C=C9)C(OC%10=C8C=CC(N)=C%10S(=O)([O-])=O)=C(S(=O)([O-])=O)C9=[NH2+])=O)N=N6)CC5)C=C4)=C1)O
**70**	O=C(C1=CC2=C(C=CC(C3=CC=C(C=C3)C(F)(F)F)=C2)C(C4=CC=C(C5CCN(C(CCCNC(CCCNC(NC6=CC7=C(C8(OC7=O)C9=C(C=C(C=C9)O)OC%10=C8C=CC(O)=C%10)C=C6)=S)=O)=O)CC5)C=C4)=C1)O
**71**	OC(C1=CC(N2N=NC(C3=CC=C(C(F)(F)F)C=C3)=C2)=CC(C4=CC=C(C5CCNCC5)C=C4)=C1)=O
**72**	O=C(C1=CC=C(C(F)(F)F)C=C1)NC2=CC(C3=CC=C(C4CCNCC4)C=C3)=CC(C(O)=O)=C2
**73**	O[C@@]1(C(C=C2)=CC=C2C3=CC(C(O)=O)=CC4=C3C=CC(C5=CC=C(C(F)(F)F)C=C5)=C4)[C@H]6CNC[C@@H]1C6
**74**	O=C(C1=CC2=C(C=CC(C3=CC=C(C(F)(F)F)C=C3)=C2)C(C4=CC=C(C5CCN(C(C)=O)CC5)C=C4)=C1)O
**75**	O=C(C1=CC(NC(C2=CC=C(C=C2)C(F)(F)F)=O)=CC(C3=CC=CO3)=C1)O
**76**	CC1=CC=C(C(NC2=CC(C(O)=O)=NN2CC3=CC=C(F)C=C3)=O)C=C1
**77**	CC1=CC=C(C(NC2=C(OC3=NC=C(F)C=C3)SC(C(O)=O)=C2)=O)C=C1
**78**	O=C(C1=CC2=C(C=CC(C3=CC=C(C=C3)C(F)(F)F)=C2)C(C4=CC=C(C5CCN(CCC6=CN([C@H]7[C@H](O)[C@@H](O)[C@H](O)[C@@H](CO)O7)N=N6)CC5)C=C4)=C1)O
**79**	O=C(C1=CC2=C(C=CC(C3=CC=C(C=C3)C(F)(F)F)=C2)C(C4=CC=C(C5=CN([C@H]6[C@H](O)[C@@H](O)[C@H](O)[C@@H](CO)O6)N=N5)C=C4)=C1)O

Compound	SMILES
**80**	CC(C(NC(C)C(O)=O)=O)C(C=C1)=CC(F)=C1C2=CC=CC=C2
**81**	COC1=CC=C(CC(NC2=CC(C3=NC4=C(O3)C=CC=C4)=CC=C2)=O)C=C1
**82**	COC1=CC=C(CC(NC2=CC(C3=NC4=C(O3)C=CC=C4)=CC=C2)=O)C=C1
**83**	O=C(NC1=CC2=C(N=CN2)C=C1)COC3=CC=C(Br)C=C3
Non-selective P2YR antagonists	
**89**	O=C(NC1=CC(C(NC2=C3C(C=C(S(=O)(O[Na])=O)C=C3S(=O)(O[Na])=O)=C(S(=O)(O[Na])=O)C=C2)=O)=CC=C1)NC4=CC(C(NC5=CC=C(S(=O)(O[Na])=O)C6=CC(S(=O)(O[Na])=O)=CC(S(=O)(O[Na])=O)=C56)=O)=CC=C4
**90**	O=P(OCC1=C(C=O)C(O)=C(C)N=C1/N=N/C2=CC=C(S(=O)(O)=O)C=C2S(=O)(O)=O)(O)O
**91**	O=C(C1=C(N)C(S(=O)(O)=O)=CC(NC2=CC=C(NC3=NC(NC4=CC(S(=O)(O)=O)=CC=C4)=NC(Cl)=N3)C(S(=O)(O)=O)=C2)=C1C5=O)C6=C5C=CC=C6
**92**	O=C1C(C2=NC=CC=C2)=[N+]([O-])C3=CC=CC=C31.O=S(C4=CC=C(C)C=C4)(O)=O
**93**	O=C(NC1=CC=CC(C(NC2=C(C)C=CC(C(NC3=CC=C(S(=O)(O)=O)C4=C3C([S])=CC(S(=O)(O)=O)=C4)=O)=C2)=O)=C1)NC5=CC(C(NC6=CC(C(NC7=C(C(S(=O)(O)=O)=CC(S(=O)(O)=O)=C8)C8=C(S(=O)(O)=O)C=C7)=O)=CC=C6C)=O)=CC=C5.OO[O]
**94**	O=C(OC1=CC=CC=C1)NC2=CC(C(NC3=CC(C(NC4=C(C(S(=O)(O)=O)=CC(S(=O)(O)=O)=C5)C5=C(S(=O)(O)=O)C=C4)=O)=CC=C3C)=O)=CC=C2
**95**	O=C(C1=CC=C(Cl)C=C1)N2C(C)=C(CC(NC(NC3=C(Cl)C=C([N+]([O-])=O)C=C3Cl)=S)=O)C4=CC(OC)=CC=C42
